# Effects of Complementary Feed with Postbiotics Derived from *Bacillus subtilis* on Nutrient Digestibility, Faecal Characteristics, Gut Microbiota, Metabolic and Immune Responses, and Coat Quality in Healthy Senior Dogs

**DOI:** 10.3390/ani16162515

**Published:** 2026-08-12

**Authors:** Oguzhan Kahraman, Martyna Wilk, Michał Bochynek, Krzysztof Bojanowski, Geovani Quijas, Zekeriya Safa Inanc, Fatma Inal, Ibrar Ahmed, Agnieszka Lewińska, Battal Yilmaz

**Affiliations:** 1 Department of Animal Nutrition and Nutritional Diseases, Faculty of Veterinary Medicine, Selçuk University, Konya 42250, Türkiye; zsafa.inanc@selcuk.edu.tr (Z.S.I.); final@selcuk.edu.tr (F.I.); 2 Department of Animal Nutrition and Feed Management, Wrocław University of Environmental and Life Sciences, 25 C.K. Norwida Street, 50-375 Wrocław, Poland; 3 BioDose, ul. Warpnowska 28, 60-453 Poznań, Poland; michal.bochynek@biodose.net; 4 Sunny BioDiscovery, Inc., 972 E. Main Street, Santa Paula, CA 93060, USA; kbojanowski@sunnybiodiscovery.com (K.B.); geovani@sunnybiodiscovery.com (G.Q.); 5 Xiamen Creciente Agritech Co., Ltd., No. 482, Xiamen 361022, China; vetrao6@gmail.com; 6 Faculty of Chemistry, University of Wrocław, Joliot-Curie 14, 50-383 Wrocław, Poland; agnieszka.lewinska@uwr.edu.pl; 7 Institute of Health Sciences, Selcuk University, Alaeddin Keykubad Campus, Yeni Istanbul Street, No:335, Konya 42130, Türkiye; vet.battalyilmaz@gmail.com

**Keywords:** *Bacillus subtilis*, blood biochemistry, senior dogs, digestibility, gut microbiota, immunity, postbiotic

## Abstract

The aim of this study was to evaluate the effects of a *Bacillus subtilis*-derived postbiotic on apparent total tract nutrient digestibility, faecal fermentation characteristics, gut microbiota, immune response, blood biochemistry, hematological parameters, serum trace minerals, and coat quality in healthy senior dogs. Twenty-one healthy senior Golden Retriever dogs (9 ± 1.0 years old) were randomly allocated to three dietary treatments for 28 days: a control diet (CON) or the same diet supplemented with 0.3 (POS1) or 0.6 mL/day (POS2) of a *Bacillus subtilis*-derived postbiotic. Apparent nutrient digestibility, faecal characteristics, blood variables, and coat quality were evaluated, whereas faecal bacterial communities were characterized by 16S rRNA gene amplicon sequencing, followed by taxonomic classification and alpha- and beta-diversity analyses. Postbiotic supplementation significantly improved crude protein and crude fiber digestibility while reducing faecal pH and ammonia concentrations and increasing acetate, propionate, and total short-chain fatty acid concentrations (*p* < 0.05). Alpha diversity was minimally affected, with a significant difference observed only for amplicon sequence variant (ASV) richness, whereas beta diversity analyses demonstrated significant alterations in overall microbial community composition. Postbiotic supplementation increased the relative abundance of *Faecalibacterium*, *Ligilactobacillus*, *Bifidobacterium*, *Prevotella*, *Turicibacter*, and *Fusobacterium*, while reducing *Bacteroides* and *Achromobacter*. Serum IgG concentrations increased, whereas cholesterol and triglyceride concentrations decreased in supplemented dogs (*p* < 0.05). Coat morphology and gloss also improved, without adverse effects on hematological, biochemical, or serum trace mineral parameters. In conclusion, *Bacillus subtilis*-derived postbiotic supplementation improved nutrient utilization, beneficially modulated faecal microbial ecology and fermentation, enhanced selected metabolic and immune responses, and improved coat quality, supporting its potential as a functional dietary strategy for promoting gastrointestinal and systemic health in healthy senior dogs.

## Simple Summary

Maintaining a healthy intestinal environment is essential for digestion, immune function, and overall health in dogs. Although probiotics and prebiotics are widely used in pet nutrition, postbiotics have attracted increasing interest because they provide beneficial microbial compounds without containing live microorganisms. In this study, we investigated whether a *Bacillus subtilis*-derived postbiotic could improve digestive and metabolic health in healthy senior dogs. Dogs receiving the postbiotic showed better digestion of protein and fiber, higher production of beneficial short-chain fatty acids, lower fecal ammonia concentrations, and favorable changes in their intestinal bacterial community. The supplement was also associated with increased blood IgG concentrations, lower cholesterol and triglyceride levels, and improved coat appearance, while no adverse effects on blood or serum trace mineral parameters were observed. These findings suggest that *Bacillus subtilis*-derived postbiotics may represent a safe and effective functional ingredient for supporting gastrointestinal health, immune function, metabolic status, and coat quality in healthy senior dogs.

## 1. Introduction

Feed additives that modulate the gut microbiota are increasingly important in animal nutrition. The gut microbiota produces various health-promoting metabolites, including bacteriocins, vitamins, short-chain fatty acids (SCFAs), organic acids, and compounds that inhibit pathogenic microorganisms. The International Scientific Association of Probiotics and Prebiotics (ISAPP) introduced the concept of postbiotics in 2021, defining them as preparations of inanimate microorganisms and/or their components that confer a health benefit. Postbiotics consist of biologically active compounds generated during microbial fermentation and released after cellular inactivation. These products contain a wide variety of microbial-derived substances, including organic acids, peptides, enzymes, proteins, and lipid fractions, as well as components originating from the microbial cell components [1].

For many years, probiotics have been used as feed additives to improve growth performance, increase nutrient absorption, modulate the gut microbiota, and support the immune system of animals [2]. Despite their well-documented health benefits, the use of live probiotic microorganisms presents several practical limitations. Unlike probiotics, postbiotics do not contain viable microorganisms. This eliminates concerns related to microbial viability, host colonization, and potential horizontal transfer of genes, including antibiotic resistance genes [3]. The viability of microorganisms in probiotic formulations may decline during storage and is difficult to predict at the end of shelf life [4]. However, postbiotics provide greater stability throughout industrial processing and prolonged storage, rendering them a more practical and reliable alternative to probiotics [5]. Additionally, postbiotics enable accurate standardization and dosage control during production [6]. Moreover, the ability of probiotics to colonize and persist in the gastrointestinal tract varies among hosts, making strain selection challenging. The effects of probiotics are also often transient, as successful colonization depends on the timing of administration [7,8].

*Bacillus subtilis* has emerged as an attractive microorganism for postbiotic production because of its ability to produce extracellular enzymes and diverse bioactive metabolites, including proteases, amylases, cellulases, other carbohydrases, organic acids, and peptides that may support gastrointestinal health [9,10]. In addition to its metabolic properties, *B. subtilis* is also widely accepted for its safety and technological suitability as a feed additive [11]. *B. subtilis* is widely used in animal nutrition, where it has been associated with improved growth performance, regulation of the gut microbiota, better regulation of the immune system, and increased resistance to disease [12].

The pet food industry is increasingly focused on functional diets that provide benefits beyond basic nutrition. Consequently, commercial dry dog foods frequently incorporate ingredients and additives intended to improve nutrient digestibility, gastrointestinal function, and microbial homeostasis [13,14]. Probiotics and prebiotics are commonly used as microbiota-targeted additives, whereas postbiotics remain relatively uncommon in commercial pet foods. Thus, postbiotics represent an emerging functional ingredient in canine nutrition, with potential benefits for digestive, immune, and metabolic health [15].

Gastrointestinal health is increasingly recognized as an integrated system involving interactions among nutrient utilization, gut microbiota, and host immune function [16]. Dietary interventions that modulate one of these components may consequently influence the others. Therefore, evaluating nutrient digestibility, faecal fermentation, microbial composition, and immune responses simultaneously provides a more comprehensive assessment of the physiological effects of postbiotic supplementation in dogs. Because postbiotics contain bioactive microbial metabolites without the requirement for viable microorganisms, they may offer several practical and biological advantages over traditional microbiota-targeted additives [17]. While the effects of probiotics and prebiotics on canine gastrointestinal health, microbial ecology, immune function, and nutrient utilization have been extensively investigated, considerably less attention has been given to postbiotics in this species. Therefore, the aim of this study was to evaluate the effects of a *Bacillus subtilis*-derived postbiotic on nutrient digestibility, faecal fermentation, gut microbiota, metabolic and immune responses, and coat quality in healthy senior dogs.

## 2. Materials and Methods

### 2.1. Ethics Statement

All experimental procedures involving animals were conducted in accordance with institutional guidelines for the care and use of animals in research. The study protocol was reviewed and approved by the Ethics Committee of the Veterinary Medicine Experimental Animals Production and Research Center, Selçuk University (Approval No. 2024/011). The dogs were housed and managed at the Prof. Dr. Hümeyra Özgen Research and Application Farm, Faculty of Veterinary Medicine, Selçuk University. Animal welfare was monitored throughout the experimental period, and every effort was made to minimize stress and discomfort.

### 2.2. Animals and Study Design

The dogs were housed individually in pens consisting of an indoor area (190 × 190 cm) and an outdoor run (510 × 230 cm) at the Dog Research Unit, Faculty of Veterinary Medicine, Selçuk University. Some dogs had been donated to the university farm and were subsequently bred and raised at the faculty’s research and application facilities for academic purposes. The remaining dogs were originally sourced from local animal shelters and later incorporated into the faculty’s research unit. These animals are housed and managed specifically for scientific research and educational purposes by faculty members and students. The animals are not private property belonging to another institution/individual/farm. Twenty-one senior Golden Retriever dogs (9 ± 1.0 years old) with an average body weight of 24.6 ± 1.2 kg were included in the trial. Before allocation, body condition score (BCS) was assessed according to the World Small Animal Veterinary Association (WSAVA) 9-point Body Condition Scoring system [18]. Dogs had mean BCS = 6.2 ± 0.4. Dogs were allocated to treatment groups using a stratified allocation procedure to achieve similar body weight, BCS and sex distribution among groups. Each dog was assigned an individual identification number (1–21), and all biological samples were labeled only with these identification codes. Laboratory personnel performing the analyses were blinded to treatment allocation throughout the analytical procedures. All female dogs included in the study were spayed, whereas all male dogs were intact. The experimental groups were defined as follows: CON, control group receiving the control food (no postbiotic supplementation); POS1, group receiving the control food supplemented with 0.3 mL/dog/day postbiotic; and POS2, group given the control food supplemented with 0.6 mL/dog/day postbiotic. The postbiotic used in the present study was a commercially available product obtained from controlled fermentation of *B. subtilis* (DeliGuard, Poznań, Poland). The postbiotic was produced by submerged batch fermentation of *Bacillus subtilis* using a yeast extract-containing culture medium. Following fermentation, the culture underwent an autolysis step involving elevated temperature to partially inactivate and partially digest bacterial cells. The material was subsequently purified by microfiltration to remove microbial cells and insoluble cell fragments, followed by ultrafiltration to concentrate soluble metabolites. According to the manufacturer, the final product contains approximately 70% water, together with organic nitrogen compounds (60 g/kg crude protein), crude fat (2.0 g/kg), crude fiber (2.0 g/kg), calcium (1.2 g/kg), sodium (21.2 g/kg), and phosphorus (0.8 g/kg). The proprietary *Bacillus subtilis* production strain was not disclosed by the manufacturer due to confidentiality. Independent microbiological testing performed by an accredited external laboratory confirmed the absence of *Salmonella* spp. in 25 g and microbial counts below the detection limit for Enterobacteriaceae, total aerobic microorganisms, yeasts, and molds (<1.0 × 10^1^ CFU/g), supporting the microbiological purity of the product. According to the manufacturing process, the postbiotic is expected to contain soluble fermentation metabolites together with bacterial cell-derived components generated during autolysis, including organic nitrogen compounds, peptides, amino acids, and cell wall fragments. However, the detailed composition of individual bioactive metabolites was not disclosed by the manufacturer because of proprietary restrictions.

The basal diet formulation and feeding management were identical for all treatment groups; the only difference among the experimental diets was the inclusion level of the *Bacillus subtilis*-derived postbiotic product. The experimental dog food did not contain any other probiotic or postbiotic additives. Dogs were fed twice daily, with the calculated daily food divided equally between the morning and evening meals. The entire daily postbiotic dose was mixed thoroughly with the morning meal and offered once daily. The dogs had been routinely fed the basal extruded diet before the start of the experiment and were therefore already adapted to the diet. No additional dietary adaptation period was implemented. The addition of the postbiotic did not affect feed acceptance, and all dogs consumed their entire daily food throughout the experimental period. The daily energy requirement of each dog was estimated according to the NRC [19]. First, the resting energy requirement (RER) was calculated and the maintenance energy requirement (MER) was then estimated by applying the appropriate maintenance factor (1.4) based on the physiological status and activity level of the dogs as follows:

RER (kcal/day) = 75 × Body weight^0.75^

MER (kcal/day) = 1.4 × RER

The daily amount of food offered to each dog was calculated by dividing the individual MER by the metabolizable energy concentration of the experimental diet (kcal/kg). Nitrogen free extract (NFE) was calculated according to the following formula:

NFE, (%DM) = 100 − (CP + EE + CF + ash)

Metabolisable energy (ME) levels of extruded dry dog foods were calculated using the following 4-step calculation formula according to NRC [19]:

1. Gross energy (GE): GE (kcal/100 g) = (5.7 × CP%) + (9.4 × EE%) + [4.1 × (NFE%+ CF%)];
2. Energy digestibility (%) = 91.2 − (1.43 × CF%);
3. Digestible energy: DE (kcal/100 g) = GE (kcal/100 g) × (energy digestibility)/100;
4. Metabolisable energy: ME (kcal/100 g) = DE (kcal/100 g) − (1.04 × CP%).

### 2.3. Extruded Dog Food and Chemical Analyses

Feedstuff materials were weighed and ground to pass through a 0.8 mm sieve. After homogenisation in the mixer, water was added to the preconditioner to obtain 25–30% moisture. The mixture was then cooked for 5 min in a DG-85 twin screw extruder at a temperature increasing from 90 °C to 135 °C in 4 stages. The moist extrudates were dried in a belt system dryer starting at 50 °C for approximately 35 min and reaching a final temperature of 110 °C. Vegetable and animal oils with added vitamins and minerals were sprayed onto hot and dried foods. Production was fixed at 250 kg of food/h. The ingredient and chemical compositions of the dry extruded food used in the experiment are given in Table 1.

The chemical composition of the experimental diet was determined by analyzing dry matter (DM), ash, crude protein (CP), crude fiber (CF), and acid-hydrolyzed ether extract (EE) using standardized procedures outlined by the Association of Official Agricultural Chemists (AOAC) [20]. Crude protein content was determined using the Kjeldahl method (AOAC Method 984.13; N × 6.25). Calcium and phosphorus concentrations of the experimental diet were determined according to AOAC [20] procedures. Feed samples were dry-ashed at 550 °C, and the ash was dissolved in nitric acid. The resulting solutions were diluted with ultrapure water, and calcium and phosphorus concentrations were quantified using inductively coupled plasma optical emission spectrometry (ICP-OES). The analytical parameters (DM, CA, CP, and CF) were also measured in faecal samples to calculate apparent total tract digestibility of nutrient coefficients. Total dietary fiber (TDF) and insoluble dietary fiber (IDF) contents of the dog food were determined using an enzymatic assay kit (Megazyme, Wicklow, Ireland). Soluble dietary fiber (SDF), expressed as a percentage of DM, was calculated as the difference between TDF and IDF values [21].

### 2.4. Determination of the Digestibility of Nutrients of Dog Food

Nutrient digestibility of the experimental dry diet, with or without postbiotic supplementation, was determined using the total faecal collection method according to AOAC [20]. The study lasted 28 days, and total faecal output from each dog was collected daily during the final three consecutive days. Throughout the collection period, each pen was inspected frequently, and all faeces were collected immediately after defecation to ensure complete recovery. Fresh faecal samples were weighed immediately after collection and stored at −18 °C until further processing. Before analysis, the samples were thawed at room temperature (23–25 °C), homogenized, and dried in a forced-air oven (VWR Venti-line, Radnor, PA, USA) at 55 °C for 48 h. The dried faecal samples were then finely ground using a laboratory mill (Retsch SM100, Haan, Germany) and passed through a 1 mm sieve to obtain a uniform particle size. Apparent total tract digestibility (ATTD) coefficients were calculated from the difference between nutrient intake and faecal nutrient excretion and expressed as a percentage of nutrient intake using the following equation:

ATTD (%) = [(nutrient intake (g) − nutrient excretion (g))/nutrient intake (g)] × 100

### 2.5. Determination of Faecal Consistency Scores

During the final three days of the experimental period, faecal consistency was evaluated daily immediately after defecation by three trained investigators. Stool quality was assessed using the five-point WALTHAM Faeces Scoring System, in which scores range from 1 (hard, dry feces) to 5 (watery diarrhea), with intermediate scores representing increasing fecal moisture and decreasing stool firmness. The investigators were blinded to dietary treatment throughout the scoring procedure [22].

### 2.6. Blood Sampling

Blood samples were collected at baseline (day 0) and at the end of the experimental period (day 28) to evaluate hematological and biochemical responses. Hematological parameters (erythrocyte, granulocyte, monocyte, lymphocyte, leukocyte) were analyzed using an automated hematology analyzer (ADVIA 120, Siemens Healthineers, Tarrytown, NY, USA). Serum was obtained by centrifugation at 3000 rpm for 15 min at 4 °C and stored at −18 °C until analysis. Serum concentrations of albumin, cholesterol, triglycerides, creatinine, and blood urea nitrogen (BUN) were determined by an automated biochemical analyzer (Hitachi 747, Tokyo, Japan) at the Veterinary Medicine Laboratory of Selçuk University. Levels of serum IgE and IgG antibodies using dog-specific IgG and IgE ELISA kits (ELK Biotechnology Co., Ltd., Wuhan, China) were measured. For trace mineral determination, 0.5 mL of serum was transferred into polypropylene tubes and combined with 1 mL of concentrated nitric acid (HNO_3_) and 0.5 mL of hydrogen peroxide (H_2_O_2_). The mixtures were incubated at 60 °C for 2 h to ensure complete digestion, followed by dilution with 2.5 mL of ultrapure water. The solutions were centrifuged at 2000 rpm for 5 min, and concentrations of cobalt, chromium, copper, iron, manganese, molybdenum, selenium, and zinc were quantified by a coupled plasma mass spectrometry (ICP-MS; Agilent 7700x, Agilent Technologies, Tokyo, Japan), as previously described by Cedeño et al. [23].

### 2.7. Determination of Short-Chain Fatty Acids, Ammonia, and pH of Feces

During the last three days of the experimental period, faecal samples were collected from each dog, yielding three samples per animal. Each sample was processed separately for the determination of SCFAs, ammonia, and pH. Fresh faecal material was diluted with distilled water at a ratio of 1:5 (*w*/*v*), homogenized by shaking, and allowed to equilibrate for 1 min at ambient laboratory temperature (24 °C). Then, faecal pH was measured using a digital pH meter [24]. Short-chain fatty acid profiles of feces collected over the final three days were determined using a gas chromatograph (Agilent 6890N, Santa Clara, CA, USA), following the procedure outlined by Abd El-Wahab et al. [25]. For SCFA analysis, 10 g of fresh feces were transferred into labeled, sealed containers and mixed with 30 mL of 16% formic acid. The resulting suspensions were thoroughly homogenized and stored at 4 °C for three days before analysis. Before chromatographic measurement, samples were centrifuged at 5000 rpm for 15 min (2 L21 centrifuge, Sigma, Osterode am Hans, Germany). Faecal ammonia concentration was determined spectrophotometrically according to the method of Weatherburn [26]. For this analysis, an additional 2 g aliquot of fresh feces was diluted 1:5 with distilled water, and a 1 mL subsample was acidified with 20 µL of sulfuric acid before measurement.

### 2.8. Faecal Microbiota Analysis

Fresh fecal samples (approximately 2 g) were collected individually from each dog on the final day of the experimental period, immediately frozen, and stored at −20 °C until analysis. Total microbial DNA was extracted, and bacterial community composition was determined by high-throughput 16S rRNA gene amplicon sequencing. Sequencing data were processed to generate amplicon sequence variants (ASVs), and taxonomic classification was performed using the Kraken2 software (version 2.1.3). Taxonomic profiles were generated at the phylum, family, and genus levels. Alpha diversity was assessed using the observed numbers of ASVs, genera, families, and phyla, together with the Shannon diversity index, Simpson diversity index (1 − D), and Pielou’s evenness index. Beta diversity was evaluated using Jaccard and Sørensen–Dice dissimilarity indices. Differences in overall microbial community composition among dietary treatments were assessed using permutational multivariate analysis of variance (PERMANOVA) with 9999 permutations. Homogeneity of multivariate dispersion was evaluated using PERMDISP, and analysis of similarities (ANOSIM) was performed as a complementary multivariate analysis. Pairwise PERMANOVA comparisons were adjusted using the Benjamini–Hochberg false discovery rate (FDR) procedure. Differences in microbial community structure were visualized by principal coordinates analysis (PCoA) and non-metric multidimensional scaling (NMDS). Relative abundances of individual bacterial taxa were subsequently subjected to statistical analysis as described in Section 2.12.

### 2.9. Morphology and Shine of the Dog’s Coat

Hairs were cut above the skin surface with Metzenbaum scissors. The process of obtaining hair samples was painless for the animals and required no analgesics or anaesthesia. The hair samples were assessed using a scanning electron microscope Hitachi S-3400N (Hitachi, Tokyo, Japan) with a Thermo Scientific (Thermo Fisher Scientific, Waltham, MA, USA) ultradry dispersive X-ray spectrometry (EDS). The surface microstructure of the cleaned hair was analyzed at an operating voltage of 10 kV and a magnification of 5–500 μm. The samples were coated with an approximately 10 nm thick Au layer using a sputtering device, Cressington 108A (Cressington Scientific Instruments, Watford, UK). Gloss measurement was performed using a micro-TRI-gloss (Byk-Gardner GMBH, Geretsried, Germany). The emitted light, incident at an angle of 0°, is reflected by the mirrors, reaching an angle of incidence of 60°, which defines the specific gloss. The measurement scale uses gloss units (GU), with a higher value corresponding to greater gloss. For gloss measurements, hair samples were aligned in a single orientation and arranged as a uniform layer. Measurements were performed by applying the detector to the sample at different angular positions.

### 2.10. Minimum Inhibitory Concentrations

The MICs were determined using a broth microdilution method in 96-well microplates according to CLSI guidelines, employing reactivated strains of *Staphylococcus aureus* ATCC25923, *Clostridium perfringens* NCTC3180, *Escherichia coli* ATCC25922, and *Salmonella* Typhimurium ATCC14028 cultivated under aerobic or anaerobic conditions at 37 °C as appropriate. The inoculum was prepared from fresh colonies, standardized to 0.5 McFarland, and diluted to 5 × 10^5^ CFU/mL in Mueller–Hinton Broth. Tested compounds were subjected to two-fold serial dilutions, mixed with the inoculum (final volume 200 µL), and incubated for 18 h. The minimum inhibitory concentration (MIC) was defined as the lowest concentration completely inhibiting visible growth, assessed spectrophotometrically based on optical density measured at 600 nm. After incubation, the samples’ dilutions were assayed with agar plates to check minimum bactericidal concentrations (MBCs). All measurements were performed in triplicate at both the technical and biological levels. For tests, three samples (24 °C, 60 °C, 75 °C) were subjected to show postbiotics’ thermal stability. Postbiotic was diluted with distilled water to 10 mL volume, placed in a 15 mL plastic tube, and left for 5 min at the tested temperature. After that, samples were left to cool down and assayed.

### 2.11. Commensal Escherichia coli K-12 Test

The aim was to determine whether the preparation exhibits general antimicrobial activity or retains at least partial specificity toward pathogenic species. Various concentrations of the preparation were incubated in a 96-well microplate using *E. coli* K-12 as a model strain representative of safe gut [27]. *Escherichia coli* (strain K-12; cat.# 155067; Carolina Biological Supply, Burlington, NC, USA) was grown in Tryptic Soy Broth (cat.# 827376; Carolina Biological Supply) from an agar plate. All cultures and experiments were performed at 36(+/−1) °C. Fresh microorganism culture was inoculated and incubated with 1:1 serial dilutions of the test material for 24 h. At the end of the incubation, bacterial viability was interrogated by measuring the OD600, followed by the MTT test [28], consisting of adding MTT solution to each well for up to 30 min. The absorbance of the blue reaction product formazan, proportional to the metabolic activity of the microorganism, was quantified with Molecular Devices microplate spectrophotometer MAX190 (San Jose, CA, USA) at 570 nm, after automatic blank subtraction, as were the OD600 measurements. Blank wells, subtracted from all measurements, contained broth without bacteria.

### 2.12. Statistical Analysis

Statistical analyses were performed using IBM SPSS Statistics version 23.0 (IBM Corp., Armonk, NY, USA). Data were initially assessed for normality using the Shapiro–Wilk test and for homogeneity of variances using Levene’s test. Normally distributed variables, including nutrient digestibility, fecal fermentation characteristics, and the relative abundances of individual bacterial taxa, were analyzed by one-way analysis of variance (ANOVA), followed by Tukey’s honestly significant difference (HSD) test for multiple comparisons. Variables that did not satisfy the assumptions of normality or homogeneity of variance were analyzed using the Kruskal–Wallis test followed by Dunn’s multiple comparison test. *p*-values obtained from bacterial taxonomic comparisons were adjusted using the Benjamini–Hochberg false discovery rate (FDR) procedure. Orthogonal polynomial contrasts were used to evaluate linear and quadratic dose–response effects of postbiotic supplementation. Stool consistency scores were analyzed using the Kruskal–Wallis test. Serum trace mineral concentrations measured on days 0 and 28 were compared within each treatment group using the paired-samples *t*-test. Hematological and serum biochemical variables that did not satisfy the assumptions of normality were analyzed using the Wilcoxon signed-rank test for within-group comparisons and the Kruskal–Wallis test followed by Bonferroni-adjusted pairwise comparisons for between-group comparisons at day 28. Results are presented as mean ± standard error of the mean (SEM) or standard deviation (SD), as indicated in the tables. Statistical significance was declared at *p* < 0.05.

## 3. Results

The postbiotic fractions derived from *B. subtilis* demonstrated exceptionally strong inhibitory activity (Table 2) and bactericidal activity (Table 3) against Gram-positive pathogens, as reflected by the very low MIC values obtained for *Clostridium perfringens* (2 ppm) and *Staphylococcus aureus* (4 ppm).

**Figure 1:**
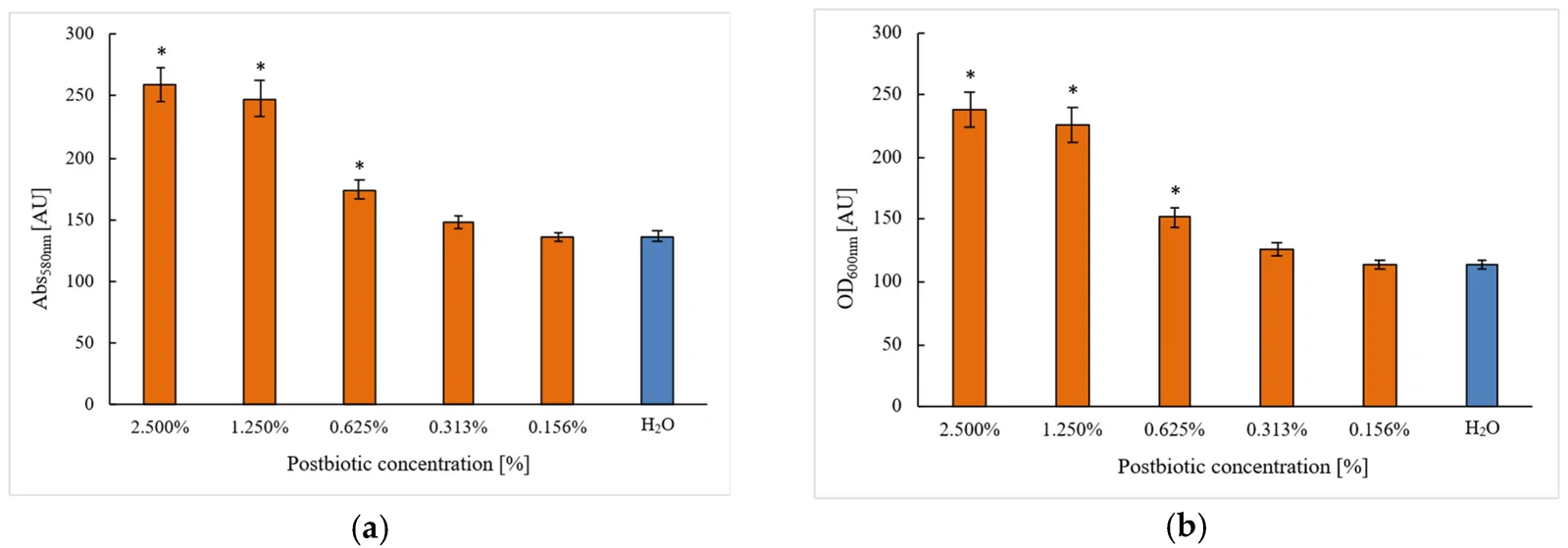
Commensal *E. coli* K-12 assay results: (**a**) MTT and (**b**) optical density readings. Asterisks show significant differences (*p* < 0.05) compared to control sample (H_2_O).

Both optical density measurements and viable cell counts using the MTT assay demonstrated that bacterial growth remained comparable to the control medium up to a concentration of 3.12 mg/mL. Moreover, increasing the concentration of the preparation in the medium resulted in a higher number of viable *E. coli* K 12 cells. At 12.5 mg/mL and 25 mg/mL, approximately a twofold increase in bacterial biomass was observed (Figure 1).

The nutrient digestibility coefficients of the groups are presented in Table 4. Dry matter digestibility (DMD) differed significantly among treatments (overall *p* = 0.044), with higher values observed in dogs receiving postbiotic supplementation compared with the control group. A similar pattern was observed for organic matter digestibility (OMD), which was significantly increased by postbiotic inclusion (overall *p* = 0.037). Crude protein digestibility (CPD) was significantly affected by dietary treatment (combined *p* = 0.008), with both postbiotic-supplemented groups exhibiting higher digestibility coefficients. Linear contrast analysis indicated a dose-dependent increase in CPD (*p* = 0.028). Crude fiber digestibility (CFD) was markedly improved by postbiotic supplementation (combined *p* = 0.001), and dogs in the POS1 and POS2 groups showed significantly higher CFD values. A significant linear effect was also detected for CFD (*p* = 0.01).

Faecal quality parameters and faecal fermentation metabolites of experimental dog groups are presented in Table 5. Faecal pH was significantly influenced by postbiotic supplementation (overall *p* = 0.007), with dogs in the POS2 group exhibiting a lower faecal pH compared to those in the control group (*p* < 0.05). A significant linear effect was observed for faecal pH (*p* = 0.001). Faecal ammonia concentration was markedly reduced in postbiotic-supplemented groups compared with the control (overall *p* = 0.001), and a significant linear decrease was detected with increasing postbiotic dose (*p* = 0.001). Postbiotic supplementation significantly affected faecal short-chain fatty acid (SCFA) profiles. Acetate and propionate concentrations were increased in dogs receiving postbiotics (combined *p* = 0.04 and 0.005, respectively), with significant linear effects observed for both parameters (*p* = 0.001 and *p* = 0.02, respectively). Total faecal SCFA concentration was significantly higher in postbiotic-supplemented groups compared with the control group (combined *p* = 0.035), and a significant linear effect was observed with increasing postbiotic dose (*p* = 0.001).

**Figure 2:**
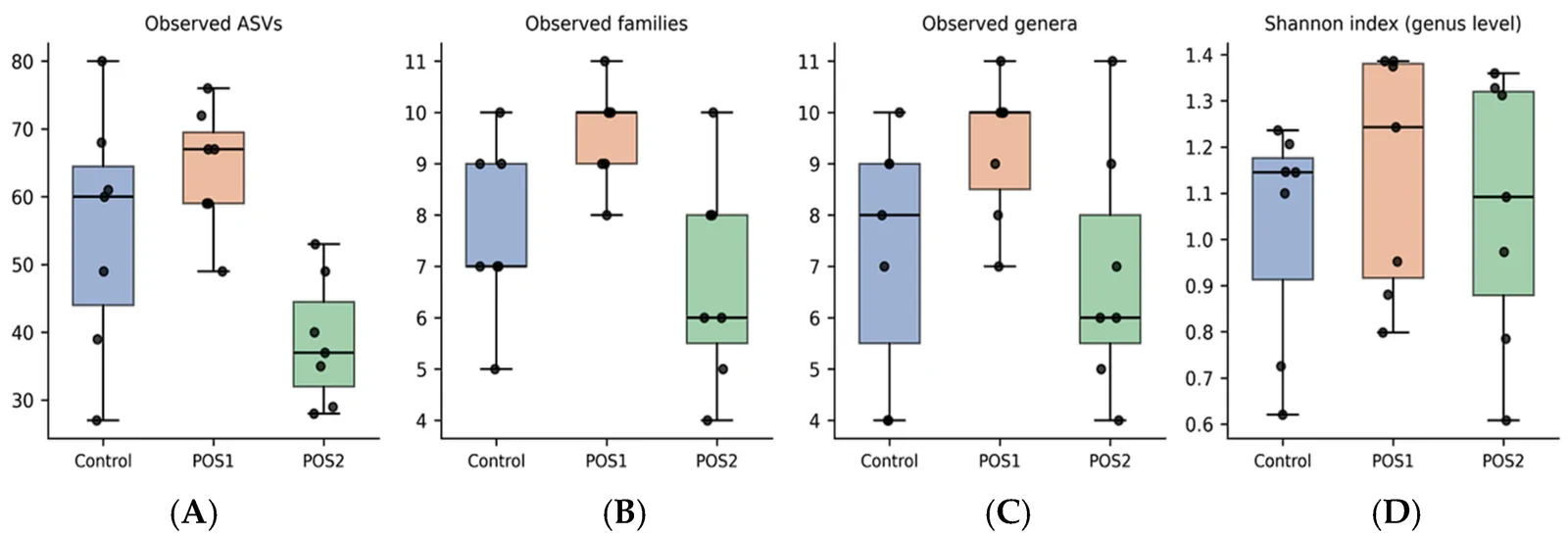
Distribution of the principal alpha diversity indices of the fecal microbiota in the Control, POS1, and POS2 groups. Boxplots show (**A**) observed ASVs, (**B**) observed families, (**C**) observed genera, and (**D**) Shannon diversity index at the genus level. Boxes indicate the interquartile range, the central line represents the median, whiskers extend to 1.5 × IQR, and dots represent individual dogs (*n* = 7).

Alpha diversity indices of the fecal microbiota are presented in Table 6. Postbiotic supplementation significantly affected bacterial richness. The observed ASV richness differed among treatment groups (FDR-adjusted *p* = 0.044), with the POS1 group exhibiting the highest richness and the POS2 group the lowest. Similarly, the observed number of bacterial families tended to differ among groups (FDR-adjusted *p* = 0.056). In contrast, the observed numbers of genera and phyla, as well as the Shannon, Simpson, and Pielou diversity indices, were not significantly affected by dietary treatment (FDR-adjusted *p* > 0.05). The boxplots presented in Figure 2 illustrate the distribution of the observed ASV richness, observed family richness, observed genus richness, and Shannon diversity index among treatment groups, supporting the statistical results presented in Table 6. The number of observed ASVs differed significantly among the experimental groups (*p* < 0.05), with dogs in the POS1 group exhibiting the highest richness, whereas the POS2 group showed the lowest number of observed ASVs. In contrast, no significant differences were detected among treatments in the number of observed families, observed genera, or the Shannon diversity index at the genus level (*p* > 0.05). Although numerical variations were observed among groups, these indices remained comparable across treatments.

**Figure 3:**
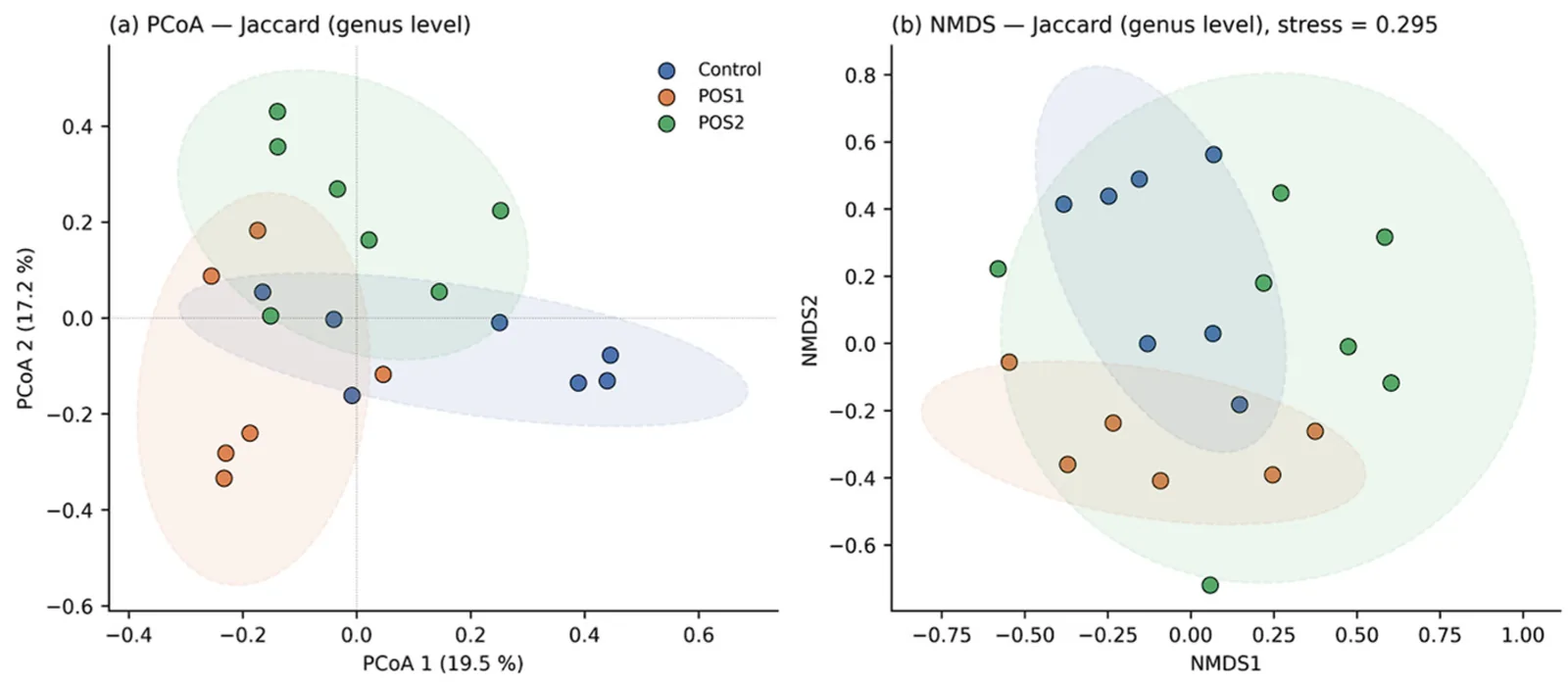
Beta diversity of the fecal microbiota based on presence/absence data at the genus level. (**a**) Principal coordinates analysis (PCoA) based on the Jaccard distance metric. The first two principal coordinates explained 19.5% and 17.2% of the total variation, respectively. (**b**) Non-metric multidimensional scaling (NMDS) based on the Jaccard distance metric (stress = 0.295). Each point represents an individual dog (*n* = 7 per group), and shaded ellipses indicate the 95% confidence intervals for each treatment group.

Beta diversity analysis revealed treatment-related differences in the fecal microbial community structure (Figure 3). Principal coordinates analysis (PCoA) based on the Jaccard distance metric demonstrated a tendency for samples to cluster according to dietary treatment, although partial overlap among treatment groups was observed. Similar clustering patterns were obtained using non-metric multidimensional scaling (NMDS), indicating consistent differences in community composition among the experimental groups.

Beta diversity analysis demonstrated significant differences in microbial community composition among dietary treatments (Table 7). PERMANOVA detected significant differences at the ASV, genus, and family levels irrespective of the dissimilarity metric used (*p* < 0.001). ANOSIM confirmed these findings (*p* < 0.001). At the ASV level, PERMDISP indicated significant heterogeneity of multivariate dispersion (*p* < 0.01), whereas no significant differences in dispersion were detected at the genus or family levels, supporting the robustness of the genus- and family-level PERMANOVA results.

Pairwise PERMANOVA analyses demonstrated significant differences in microbial community composition between all treatment groups (Table 8). At the ASV and genus levels, all pairwise comparisons remained significant after Benjamini–Hochberg correction (FDR-adjusted *p* ≤ 0.004). At the family level, significant differences were also observed among all treatment groups (FDR-adjusted *p* ≤ 0.035). These results indicate that both postbiotic supplementation levels altered the fecal microbial community relative to the control group and that the microbial community composition also differed significantly between the two postbiotic doses.

The relative abundances of the dominant bacterial phyla are presented in Table 9. Postbiotic supplementation significantly affected the phylum-level composition of the fecal microbiota. The relative abundance of Firmicutes increased significantly in postbiotic-supplemented dogs compared with the control group (FDR-adjusted *p* = 0.037), whereas Bacteroidota decreased significantly following supplementation (FDR-adjusted *p* = 0.045). No significant differences were observed for Fusobacteriota, Proteobacteria, Actinobacteriota, or Campylobacterota after correction for multiple comparisons (FDR-adjusted *p* > 0.05).

The relative abundances of the major bacterial genera are presented in Table 10. Dietary supplementation with the *Bacillus subtilis*-derived postbiotic significantly affected the abundance of several bacterial genera. Compared with the control group, both postbiotic-supplemented groups showed significantly higher relative abundances of *Turicibacter* and *Ligilactobacillus* (*p* < 0.05). The abundance of *Faecalibacterium* was also increased in both supplemented groups, with the highest value observed in the POS1 group (*p* = 0.008). The relative abundances of *Bifidobacterium*, *Segatella*, *Streptomyces*, and *Fusobacterium* increased in a dose-dependent manner, whereas *Bacteroides* and *Achromobacter* decreased following postbiotic supplementation. In contrast, *Sarcina* was significantly reduced in both supplemented groups compared with the control group (*p* < 0.05). *Enterococcus* and *Streptococcus* reached their highest relative abundances in the POS1 group, whereas *Prevotella* was highest in the POS2 group. No significant differences were observed for *Clostridium*, *Neisseria*, *Terrisporobacter*, or *Lactobacillus* among the dietary treatments (*p* > 0.05).

**Figure 4:**
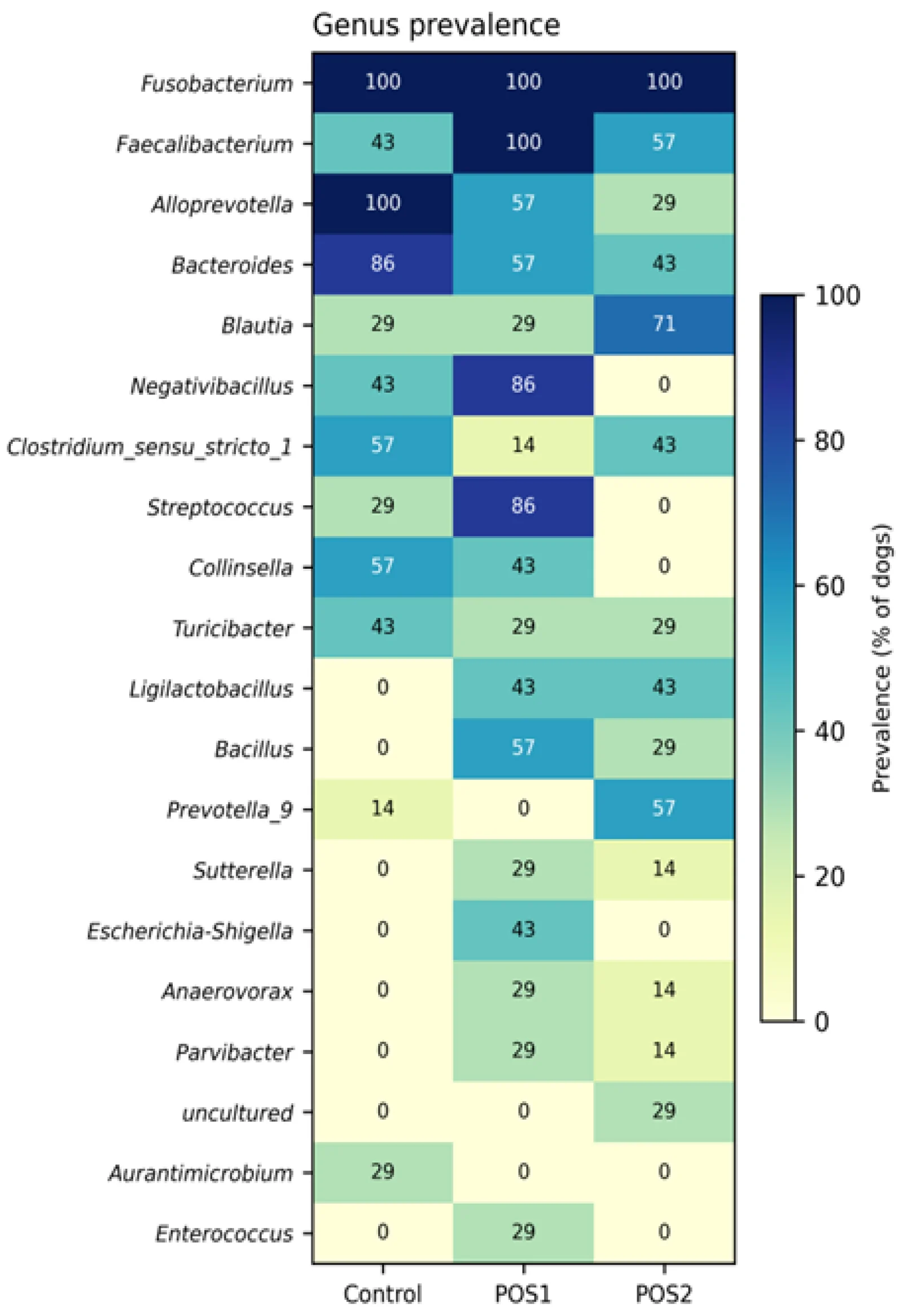
Heatmap illustrating the prevalence of the major bacterial genera in the fecal microbiota of dogs fed the experimental diets. Each cell represents the percentage of dogs within a treatment group in which the corresponding bacterial genus was detected. The color gradient ranges from light yellow (low prevalence) to dark blue (high prevalence), indicating increasing prevalence across samples.

The prevalence heatmap of the major bacterial genera is presented in Figure 4. Distinct prevalence patterns were observed among treatment groups. *Fusobacterium* was detected in all dogs (100%) regardless of dietary treatment. Several genera, including *Faecalibacterium*, *Bacteroides*, *Blautia*, *Negativibacillus*, *Streptococcus*, *Ligilactobacillus*, *Bacillus*, and *Prevotella_9*, exhibited treatment-dependent differences in prevalence, supporting the alterations in microbial composition observed in the relative abundance analysis. While *Fusobacterium* was consistently detected in all dogs, other genera exhibited variable prevalence according to dietary treatment.

**Figure 5:**
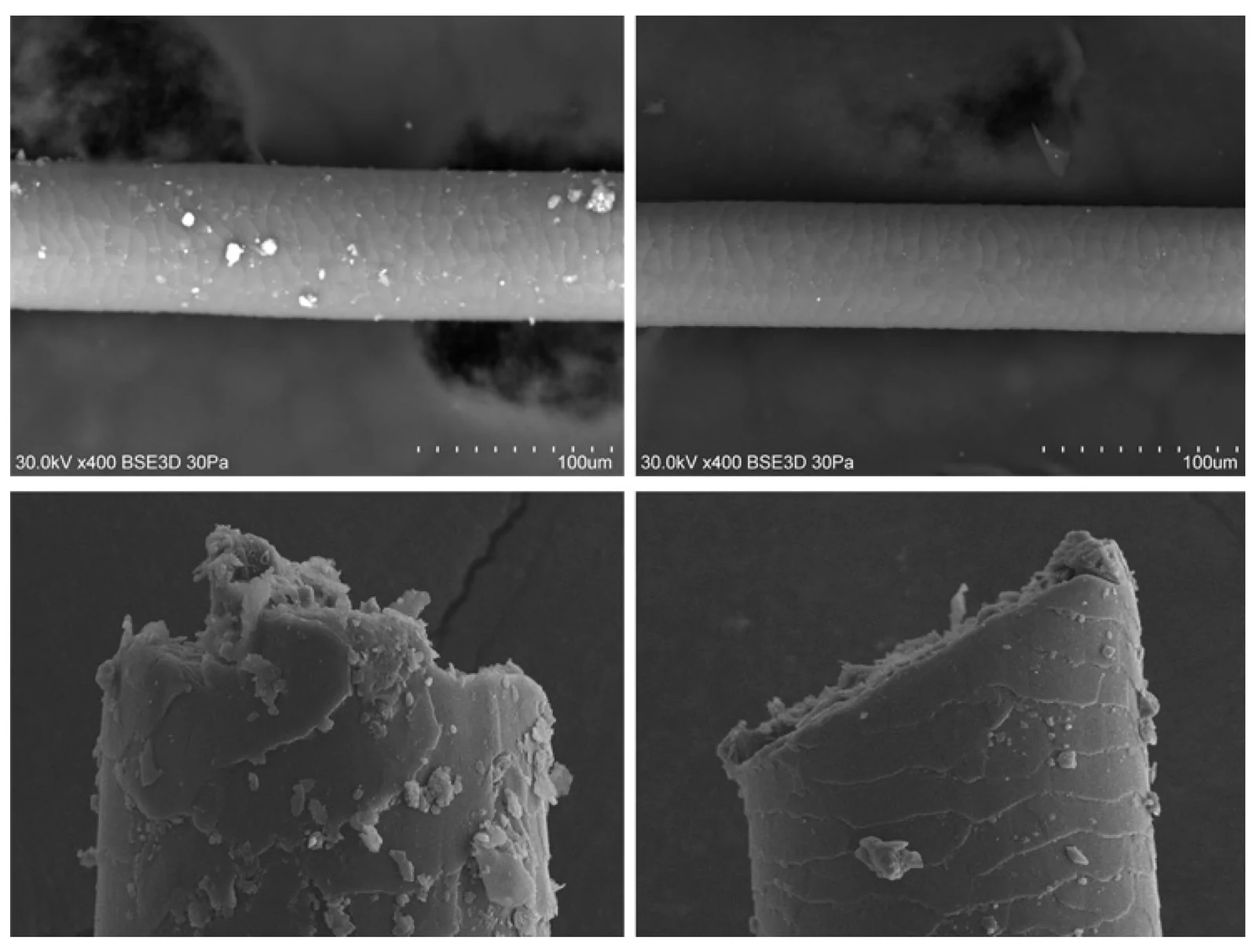
SEM imaging of hair at two magnifications before (**left**) and after (**right**) postbiotic administration.

Scanning electron microscopy (SEM) revealed clear differences in hair surface morphology between baseline and postbiotic-supplemented samples (Figure 5). In the control condition (day 0), hair fibers exhibited irregular cuticle structures, with visible surface debris and discontinuities along the shaft. In contrast, hair samples obtained after postbiotic supplementation showed a more uniform and smoother surface, with fewer adherent particles and improved cuticle integrity. At higher magnification, hair tips in the control samples appeared fragmented and uneven, whereas postbiotic-treated samples displayed more continuous and structurally organized cuticle edges.

Biochemical and hematological parameters measured at day 0 and day 28 are presented in Table 11. Within-group comparisons using the Wilcoxon signed-rank test revealed a significant increase in monocyte counts in the POS2 group between day 0 and day 28 (*p* = 0.018). In addition, significant time-dependent changes were observed in the postbiotic-supplemented groups for selected erythrocyte indices and biochemical variables. No significant effects of postbiotic supplementation were observed for serum IgE concentrations during the experimental period (*p* > 0.05). In the POS1 group, mean corpuscular hemoglobin concentration (MCHC), immunoglobulin G (IgG), creatinine, cholesterol, and triglyceride levels differed significantly over time (*p* < 0.05). Similarly, in the POS2 group, significant changes between day 0 and day 28 were detected for mean corpuscular volume (MCV), MCHC, IgG, creatinine, cholesterol, and triglyceride concentrations (*p* < 0.05). Between-group comparisons performed at day 28 using Bonferroni-adjusted tests indicated significant differences among CON, POS1, and POS2 groups for MCV, blood urea nitrogen (BUN), cholesterol, and triglyceride concentrations (*p* < 0.05).

Postbiotic supplementation resulted in significant time-dependent changes in several trace minerals (Table 12). In the POS1 group, serum iron, manganese, and selenium concentrations were significantly increased at day 28 compared with baseline values (*p* < 0.05). Similarly, dogs in the POS2 group exhibited significant increases in serum copper, chromium, iron, and manganese concentrations over the experimental period (*p* < 0.05).

**Figure 6:**
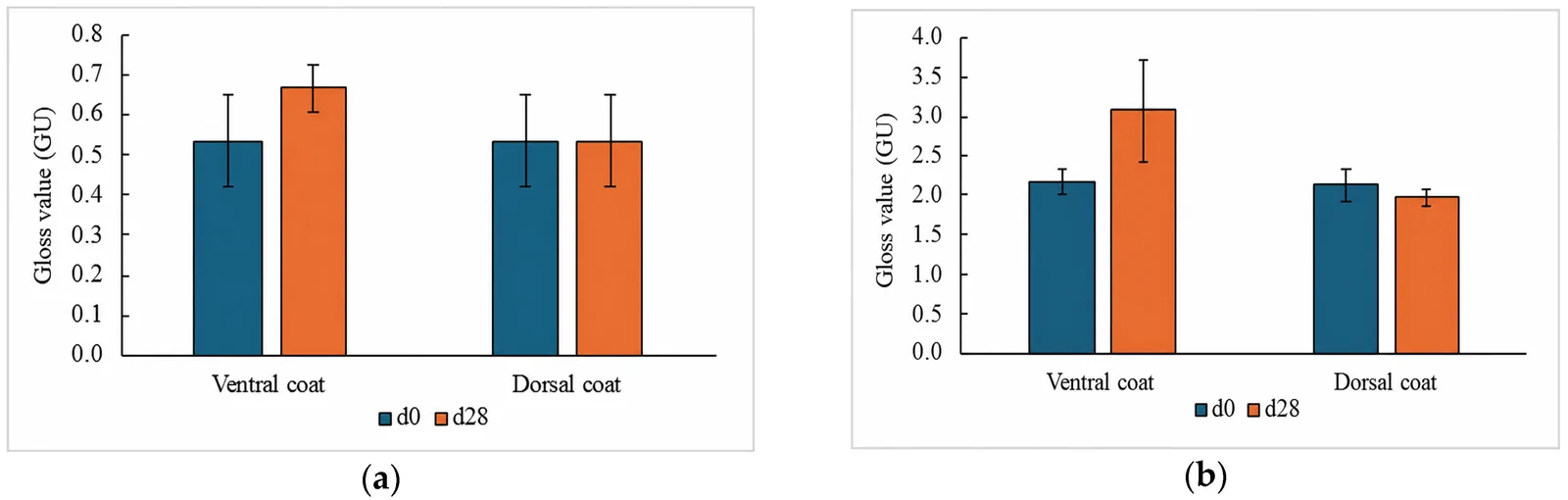
Comparison of gloss values obtained for samples before and after postbiotic treatment at measurement angles of (**a**) 20° and (**b**) 60°.

Hair gloss measurements are presented in Figure 6. Visual examination of the measurements suggested a tendency toward higher gloss values following postbiotic supplementation. This tendency was observed at both 20° and 60° measurement angles and appeared more pronounced in the ventral region than in the dorsal region. Because no formal statistical analysis was performed, these observations should be interpreted descriptively.

## 4. Discussion

Aging in dogs is associated with alterations in gastrointestinal physiology, including compromised intestinal barrier function and changes in gut microbiota composition, which may collectively impair nutrient utilization [29]. The dogs enrolled in the present study were classified as healthy senior animals (9–10 years old), an age at which physiological changes in gastrointestinal function and microbial ecology become increasingly evident. Therefore, the greater improvements in organic matter, dry matter, crude protein, and crude fiber digestibility observed following postbiotic supplementation may reflect the greater responsiveness of the aging gastrointestinal tract to microbial-derived bioactive compounds, thereby contributing to improved digestive efficiency in senior dogs.

Because all dogs received the same basal extruded diet throughout the study, dietary composition was consistent across treatment groups, allowing for differences among groups to be interpreted in the context of postbiotic supplementation while recognizing the contribution of the common dietary background. Diet composition, particularly dietary fiber and fermentable substrates, is one of the major determinants of gut microbial composition and fermentation activity in dogs [13]. Nevertheless, the basal diet itself, particularly its fiber sources and other fermentable substrates, likely contributed to intestinal fermentation and supported the resident gut microbiota. Therefore, the microbiota and fermentation responses observed in the present study should not be attributed exclusively to postbiotic supplementation. Rather, the postbiotic should be considered as a dietary intervention acting within the context of a common basal diet, potentially modulating microbial activity and fermentation processes already supported by the dietary substrate. It also should be noted that the metabolizable energy value reported for the experimental diet was estimated using the NRC (2006) [19] predictive equations rather than determined experimentally through metabolizable energy trials. Therefore, the observed improvements in nutrient digestibility should not be interpreted as direct evidence of changes in dietary metabolizable energy.

The present study demonstrated a dose-dependent improvement in apparent total tract digestibility of dry matter, organic matter, crude protein, and crude fiber following supplementation with the *Bacillus subtilis*-derived postbiotic, with the most pronounced responses observed for crude protein and crude fiber digestibility. Several mechanisms may explain the improved digestibility observed in the present study. According to the manufacturer’s information, the postbiotic was produced through submerged fermentation followed by autolysis and membrane filtration, resulting in a preparation rich in soluble microbial metabolites and organic nitrogen-containing compounds rather than viable bacterial cells. These soluble components, together with bacterial cell-derived molecules generated during autolysis, may enhance digestive processes by modulating gastrointestinal physiology, stimulating digestive secretions, or improving intestinal epithelial function. In addition, *B. subtilis* is well-recognized for producing extracellular enzymes during fermentation, including proteases, amylases, and fibrolytic enzymes [30]. Although enzyme activity was not quantified in the present product, residual enzymatic activity or fermentation-derived metabolites may have contributed to the improved degradation of dietary protein and fiber, thereby increasing nutrient availability. The biological relevance of these improvements is particularly noteworthy because the experimental animals were healthy senior dogs, in which age-related reductions in digestive efficiency and intestinal function have previously been described. In contrast, Wren et al. [31] reported that postbiotic supplementation did not significantly affect apparent nutrient digestibility in healthy adult dogs. The discrepancy between their findings and those of the present study may be attributable to differences in the source and composition of the postbiotic, supplementation dose, duration of feeding, dietary composition, or the physiological characteristics of the animals. The *Bacillus subtilis*-derived postbiotic used in the present study may have contained a distinct profile of bioactive metabolites capable of promoting microbial fermentation and enhancing digestive efficiency.

Postbiotics encompass a broad spectrum of biologically active molecules, including short-chain fatty acids (SCFAs), bacteriocins, enzymes, exopolysaccharides, peptidoglycans, lipoteichoic acids, and other cell-wall-derived components generated during microbial fermentation or inactivation processes [32]. While some bioactive compounds, such as organic acids, may remain stable throughout gastrointestinal transit, others may undergo partial degradation into smaller fragments that may still retain biological activity [33]. The changes observed here probably resulted from the combined effects of postbiotic-derived bioactive compounds on the intestinal ecosystem. Enhanced fiber digestibility may have increased the availability of fermentable substrates for saccharolytic bacteria, while the accompanying shifts in microbial composition likely promoted a fermentation pattern characterized by greater acetate and propionate production and lower ammonia accumulation. Collectively, these changes suggest a more efficient utilization of dietary nutrients and a transition toward a metabolically favorable intestinal environment.

The concomitant decrease in fecal ammonia together with the increase in acetate and propionate suggest a shift in microbial metabolism from proteolytic toward saccharolytic fermentation. This interpretation is further supported by the improved crude fiber digestibility, which may have increased the availability of fermentable carbohydrate substrates for colonic microorganisms. Greater utilization of carbohydrates generally favors SCFA production while reducing amino acid deamination and ammonia generation in dogs [34]. Importantly, despite these metabolic changes, fecal consistency score and fecal output remained unchanged, indicating that postbiotic supplementation modified microbial fermentation without adversely affecting stool quality. This observation suggests that the postbiotic primarily influenced intestinal metabolic activity rather than causing clinically relevant alterations in bowel function, supporting its potential suitability as a functional dietary ingredient for healthy senior dogs. This observation is consistent with previous reports indicating that postbiotics and fermentable substrates primarily modulate microbial metabolism and fermentation patterns without necessarily altering fecal characteristics, suggesting that metabolic adaptations may precede measurable changes in stool quality [35]. Similar to the present study, de Souza Junior et al. [36] reported that a heat-treated postbiotic form of *Bifidobacterium animalis* positively affected the intestinal health of cats. Daily supplementation with this postbiotic resulted in higher faecal propionate concentrations, a SCFA known to support gut health and exert multiple beneficial physiological effects. Furthermore, in vitro supplementation of dog faecal samples with postbiotics has been reported to promote the growth of beneficial bacterial populations associated with elevated faecal acetate, propionate, and butyrate production [37]. The observed increase in fecal acetate, propionate, and total SCFAs is likely multifactorial. It may reflect the combined effects of bioactive compounds present in the postbiotic preparation, together with postbiotic-induced modulation of gut microbial communities toward SCFA-producing taxa. In addition, digestive enzymes, bioactive peptides, and oligosaccharides generated during fermentation may have increased the availability of fermentable substrates, whereas changes in luminal pH, mucus integrity, and host–microbial interactions may have further promoted microbial SCFA production. Collectively, these findings indicate that *Bacillus subtilis*-derived postbiotics promote a more metabolically favorable intestinal environment characterized by enhanced saccharolytic fermentation and reduced protein fermentation, which may contribute to improved intestinal health in senior dogs.

The metabolic changes observed in the present study were accompanied by measurable alterations in the fecal microbial community, suggesting that *Bacillus subtilis*-derived postbiotic supplementation influenced not only microbial metabolic activity but also the ecological structure of the gut microbiota. Because microbial composition and microbial metabolism are closely interconnected, the changes in SCFA production, ammonia concentration, and nutrient digestibility observed in the present study are likely to be associated, at least in part, with shifts in the intestinal microbial community. Although postbiotic supplementation significantly affected bacterial richness, its influence on overall microbial diversity was relatively limited. The observed ASV richness differed among dietary treatments, whereas Shannon, Simpson, and Pielou diversity indices remained unchanged. These findings indicate that the postbiotic primarily reshaped the composition of the gut microbiota rather than substantially altering its overall diversity. Similar observations were reported by Wren et al. [31], who observed that supplementation with a *Lacticaseibacillus paracasei*-derived postbiotic or live *Bacillus pumilus* modified the abundance of selected bacterial taxa while exerting minimal effects on overall fecal microbial diversity in healthy adult dogs. In contrast, Kayser et al. [38] reported increased bacterial richness together with changes in selected alpha diversity indices following 90 days of supplementation with a heat-treated *Bifidobacterium animalis* subsp. *lactis* postbiotic. Consistent with this, the systematic review by Bonel-Ayuso et al. [1] concluded that postbiotic supplementation generally exerts limited effects on alpha diversity in dogs, suggesting that detectable changes may depend on factors such as supplementation duration, host characteristics, and the type of postbiotic used.

Because all dogs included in the present study were healthy senior animals of a similar age (9–10 years), age-related variation in gut microbial diversity was expected to be minimal. Aging has been associated with gradual alterations in the intestinal microbiota, including shifts in the relative abundance of specific bacterial taxa, reduced microbial resilience, and changes in microbial metabolic activity. However, previous studies have shown that these age-related changes are generally characterized by subtle alterations in microbial composition rather than substantial reductions in overall microbial diversity [39]. Consequently, the relatively homogeneous age distribution of the dogs in the present study minimizes age as a potential confounding factor. Therefore, the observed differences in bacterial community composition and the concurrent changes in SCFA production, fecal ammonia concentration, and nutrient digestibility are more likely related to postbiotic supplementation than to age-related variation in the gut microbiota.

In contrast to the relatively limited effects on alpha diversity, beta diversity analyses demonstrated that postbiotic supplementation altered the overall structure of the fecal microbial community. Although partial overlap among treatment groups remained, PCoA and NMDS ordination analyses together with PERMANOVA and ANOSIM indicated treatment-related shifts in microbial community composition. These findings suggest that the postbiotic selectively modified the ecological organization of the gut microbiota rather than broadly increasing microbial diversity. Similar observations were reported by Wren et al. [31], who found that supplementation with either a *Lacticaseibacillus paracasei*-derived postbiotic or live *Bacillus pumilus* modified the abundance of selected bacterial taxa while exerting minimal effects on overall fecal microbial diversity in healthy adult dogs. Likewise, the systematic review by Bonel-Ayuso et al. [1] concluded that postbiotic supplementation generally has limited effects on alpha diversity in dogs despite consistent alterations in microbial composition. In contrast, Kayser et al. [38] reported increased richness together with modest changes in selected alpha diversity indices following 90 days of supplementation with a heat-treated *Bifidobacterium animalis* postbiotic. Therefore, the 28-day intervention may have been sufficient to induce selective shifts in microbial community composition, as evidenced by the significant PCoA, NMDS, PERMANOVA, and ANOSIM results, but insufficient to produce broader changes in alpha diversity.

In contrast to alpha diversity, beta diversity analyses demonstrated treatment-related differences in microbial community structure. Although partial overlap among groups remained, PCoA, NMDS, PERMANOVA and ANOSIM collectively indicated that postbiotic supplementation modified the overall composition of the fecal microbiota. These findings suggest that the postbiotic selectively modified the microbial community structure rather than broadly increasing microbial diversity. Similar observations have been reported following postbiotic supplementation in dogs, where changes in microbial community composition occurred despite relatively limited alterations in global alpha diversity. Moreover, a recent systematic review and meta-analysis concluded that the current evidence regarding the effects of postbiotics on canine gut microbiota remains limited because of small sample sizes, heterogeneous postbiotic formulations, and differences in study design and supplementation duration Bonel-Ayuso et al. [1]. Therefore, variations among studies are likely to reflect methodological differences rather than contradictory biological effects.

At the genus level, postbiotic supplementation selectively enriched several bacterial taxa associated with intestinal homeostasis and saccharolytic metabolism. Among these, the increased abundances of *Faecalibacterium*, *Bifidobacterium*, and *Ligilactobacillus* are particularly noteworthy because these genera play complementary roles in maintaining intestinal function. *Faecalibacterium* is one of the principal short-chain fatty acid (SCFA)-producing bacteria in the canine intestine and is widely associated with anti-inflammatory activity and intestinal homeostasis. In contrast, *Bifidobacterium* and *Ligilactobacillus* primarily contribute to carbohydrate fermentation, organic acid production, and maintenance of epithelial barrier integrity, while also providing metabolic substrates that support cross-feeding interactions with other SCFA-producing bacteria. Accordingly, the enrichment of these genera provides a biologically plausible explanation for the increased fecal acetate, propionate, and total SCFA concentrations, together with the reduced fecal ammonia levels, observed in the present study. Similarly, Kayser et al. [38] reported that supplementation of healthy dogs with a heat-treated *Bifidobacterium animalis* postbiotic selectively modulated the intestinal microbiota and increased fecal propionate concentrations without markedly altering overall microbial diversity. Furthermore, a recent systematic review by Bonel-Ayuso et al. [1] summarized several canine studies reporting enrichment of beneficial genera, including *Faecalibacterium* and *Bifidobacterium*, following postbiotic supplementation. Collectively, these findings indicate that postbiotics primarily improve intestinal function through selective remodeling of metabolically beneficial bacterial populations rather than by inducing broad changes in overall microbial diversity. This interpretation is further supported by the concomitant improvements in nutrient digestibility, increased fecal SCFA concentrations, and reduced fecal ammonia observed in the present study.

The increased abundances of *Prevotella* and *Turicibacter* further support enhanced saccharolytic fermentation in postbiotic-supplemented dogs. *Prevotella* species possess extensive enzymatic capacity for degrading complex carbohydrates and dietary fiber, thereby contributing to propionate production and improved utilization of fermentable substrates Bonel-Ayuso et al. [1]. Consistent with this role, the increased abundance of *Prevotella* coincided with higher crude fiber digestibility and elevated fecal propionate concentrations in the present study. Koziol et al. [40] demonstrated that supplementation with a *Lactobacillus* fermentation product modified the fecal microbiota and altered microbial metabolic activity toward saccharolytic fermentation without significantly increasing *Prevotella* abundance, suggesting that improvements in carbohydrate fermentation may occur through different microbial pathways depending on the composition of the postbiotic product and the resident gut microbiota. In contrast, the enrichment of *Prevotella* observed in the present study may indicate that the *Bacillus subtilis*-derived postbiotic selectively favored bacterial populations specialized in complex carbohydrate utilization. *Turicibacter* has also been associated with intestinal homeostasis and healthy microbial ecosystems in dogs. A previous canine study similarly reported increased *Turicibacter* abundance following postbiotic (Saccharomyces cerevisiae-derived) supplementation, suggesting that this genus may serve as an indicator of a favorable intestinal microbial environment [41]. Likewise, the increased abundance of *Fusobacterium* observed in the present study should not be interpreted as evidence of dysbiosis, as this genus represents a common and abundant member of the healthy canine gut microbiota. Previous canine studies have likewise reported maintenance or enrichment of *Fusobacterium* following postbiotic supplementation, together with increases in other beneficial bacterial genera, indicating that different postbiotic formulations may promote favorable intestinal remodeling despite targeting distinct bacterial taxa [1].

In contrast to the enrichment of beneficial fermentative bacteria, postbiotic supplementation reduced the relative abundances of *Bacteroides*, *Achromobacter*, and *Sarcina*, indicating selective ecological remodeling rather than indiscriminate changes in the intestinal microbiota. Although *Bacteroides* constitutes an important component of the healthy canine gut microbiota, previous canine studies have not consistently reported alterations in its abundance following postbiotic supplementation. For example, Wren et al. [31] and Koziol et al. [40] observed beneficial effects of postbiotic supplementation on intestinal health without significant changes in *Bacteroides*, suggesting that taxonomic responses may vary according to the composition and bioactive metabolites of individual postbiotic formulations. Therefore, the reduction in *Bacteroides* observed in the present study is more likely to reflect altered substrate utilization and microbial competition than a generalized loss of beneficial bacteria, particularly because it occurred concurrently with increased SCFA production, improved nutrient digestibility, and reduced fecal ammonia concentrations. Likewise, the marked decline in *Achromobacter*, an opportunistic environmental bacterium that has occasionally been associated with intestinal dysbiosis and gastrointestinal disorders, may represent a favorable ecological shift rather than an adverse microbial response. A similar interpretation may apply to *Sarcina*, whose overgrowth has primarily been reported under pathological gastrointestinal conditions rather than in healthy dogs. Collectively, these findings suggest that the *Bacillus subtilis*-derived postbiotic promoted a functionally beneficial restructuring of the intestinal microbial ecosystem by suppressing bacterial taxa with limited or potentially undesirable ecological roles while enriching metabolically advantageous bacterial populations.

All measured blood biomarkers remained within physiological reference intervals. Therefore, although several biomarkers differed significantly among groups, all values remained within physiological reference intervals and should be interpreted as physiological variations rather than clinically meaningful improvements in health status. The results of hematological parameters, including WBC, RBC, HGB, HCT, and erythrocyte indices (MCV, MCH, and MCHC), indicate that postbiotic supplementation, even at the higher dose (0.6 mL/day), did not negatively affect the hematopoiesis or systemic health status of the dogs. Immunological parameters demonstrated a more pronounced response to postbiotic supplementation. In particular, serum IgG concentrations were significantly increased in postbiotic groups, especially in POS2, suggesting an enhancement of humoral immune activity. The increase in serum IgG may be associated with the immunomodulatory activity of bioactive postbiotic metabolites together with the enrichment of SCFA-producing bacterial genera, as microbial metabolites promote B-cell differentiation and antibody production [42,43]. Although conducted in a different animal species, a previous study reported that dietary supplementation with 0.3% *Lactobacillus plantarum*-derived postbiotics significantly increased serum IgA and IgM concentrations but did not affect serum IgG levels in growing mink, supporting the immunomodulatory potential of postbiotics across animal species [44]. Research on *Lactobacillus*-based postbiotics has reported inconsistent effects on humoral immunity, with some studies demonstrating significant increases in serum IgG concentrations [45], whereas others have reported no significant effects on systemic immunoglobulin levels [46]. This inconsistency may be attributed to differences in bacterial strains, dosage, preparation methods, animal species, and experimental conditions.

Postbiotic supplementation significantly reduced serum cholesterol and triglyceride concentrations, particularly in dogs receiving the higher dose (POS2). These findings are consistent with previous studies demonstrating that postbiotic-derived microbial metabolites can beneficially modulate host lipid metabolism. Seikh et al. [47] reported that dietary supplementation with inactivated *Lactobacillus* DH42 reduced serum cholesterol concentrations in broiler chickens, while Monika et al. [48] observed significant reductions in both cholesterol and triglyceride concentrations following supplementation with a *Lactobacillus acidophilus*-derived postbiotic. Although evidence in dogs remains limited, the present findings suggest that *Bacillus subtilis*-derived postbiotics may exert similar hypolipidemic effects across animal species. The observed reduction in circulating lipids may also be associated with the concurrent enrichment of SCFA-producing bacterial genera and increased fecal SCFA concentrations observed in the present study. Short-chain fatty acids, particularly propionate, have been shown to regulate hepatic lipid metabolism, suppress cholesterol synthesis, and reduce intestinal lipid absorption, thereby providing a biologically plausible mechanism linking postbiotic-induced microbial modulation with improved host lipid metabolism [49]. Collectively, these findings indicate that the metabolic benefits of postbiotic supplementation may extend beyond improvements in intestinal health to include favorable systemic effects on lipid homeostasis.

Postbiotic supplementation induced modest but significant changes in several circulating trace minerals, including copper, chromium, iron, manganese, and selenium, while all values remained within established physiological reference ranges. These findings suggest that the *Bacillus subtilis*-derived postbiotic did not disrupt mineral homeostasis but may have improved mineral utilization or bioavailability. Although evidence regarding the effects of postbiotics on trace mineral status in dogs remains limited, the present findings indicate that postbiotic supplementation may positively influence micronutrient metabolism without adversely affecting mineral homeostasis. Enhanced nutrient digestibility and increased microbial fermentation and SCFA production observed in the present study may have contributed to improved intestinal mineral absorption by lowering luminal pH and increasing mineral solubility. Previous studies have likewise suggested that beneficial modulation of the intestinal microbiota can improve the bioavailability of trace minerals through enhanced intestinal function and microbial metabolism [49].

Cell-free supernatants of *B. subtilis* BS-Q exhibit potent anti-clostridial activity, with MIC values of approximately 33 µg/mL against multiple *C. perfringens* types [50]. Similarly, secondary metabolites of *B. subtilis* HSFI-9 have been reported to inhibit *S. aureus* at remarkably low concentrations (MIC 3.125 µg/mL), whereas their efficacy against Gram-negative *Escherichia coli* is substantially lower (MIC 25 µg/mL), underscoring the inherent susceptibility differences between Gram-positive and Gram-negative bacteria [51]. These observations are consistent with the structural disparities between the two bacterial groups. Gram-positive organisms possess a thick, porous peptidoglycan layer that facilitates penetration of antimicrobial peptides and lipopeptides, whereas the outer lipopolysaccharide membrane in Gram-negative bacteria creates an effective permeability barrier. Supporting this, metabolite fractions from *B. subtilis* 6D1 have been shown to potently inhibit *S. aureus* biofilm formation and virulence, but their activity is far less pronounced against Gram-negative species [52]. Taken together, the current findings strongly reinforce the notion that *B. subtilis*-derived postbiotics preferentially target Gram-positive bacteria and exhibit limited efficacy against Gram-negative enteric pathogens such as *E. coli* and *Salmonella*, for which MIC values in our study exceeded 2 mg/mL. Thermal resistance tests showed high stability of the postbiotic mixture: samples exposed to both 60 °C and 75 °C showed the same results as the non-incubated reference (24 °C). Similar observations were reported in studies investigating the antifungal activity of secondary metabolites produced by *Bacillus* spp., where the inhibition zones against Aspergillus niger remained comparable across samples incubated at –20, 4, 25, 37, 60, 80, and 100 °C [53]. Likewise, assessments of the antiviral activity of Bacillus-derived postbiotics incubated at –20, 20, and 60 °C demonstrated that these compounds retain their bioactivity despite exposure to a wide range of temperatures, confirming their heat resistance [54].

In Golden Retrievers, hair fibers have a diameter ranging from 45 μm to 90 μm, depending on the location, and the cuticle is of the squamous type. Similar findings to those in our study were reported by Inamdar et al. [55]: at low magnification, the unsupplemented hair surface showed numerous adherent particles or deposits distributed along the cuticle surface. These may represent fragmented cuticle elements or external contaminants. In contrast, images of the supplemented dog’s fur showed a visibly cleaner and more uniform surface with markedly fewer adherent particles, indicating reduced adhesion of undesirable material. At higher magnification, the hair tips in the baseline samples exhibited irregular, jagged edges with visible surface debris and discontinuities, indicating cuticle disruption and/or surface fragmentation. After postbiotic administration, the hair tips appeared more structurally coherent, with smoother, more continuous edges and fewer surface contaminants.

Furthermore, modulation of immune system overactivity may reduce hypersensitivity reactions and skin inflammation (e.g., itching, redness), which directly translates into reduced hair breakage and loss, and improved coat appearance. The improvements observed at the level of skin and hair structure may represent a downstream manifestation of the combined effects of enhanced nutrient digestibility, microbiota modulation, and reduced intestinal proteolytic activity, all of which are supported by the stability and bioactivity of the postbiotic preparation.

The visible effect of improved condition is reflected in the structure and gloss of the animal’s coat, which indicates vitality and proper nutrition. However, visual perception depends on the observer, lighting, viewing conditions, and the properties of the object and material being examined. In this study, a comparative analysis was conducted before and after postbiotic administration, with measurements taken at three angles: 20°, 60°, and 85°, corresponding to high, medium, and low gloss, respectively. Due to very low values with high variability obtained at the 85° angle, only measurements at 20° and 60° were included in the analysis. The results (Figure 6) showed a trend toward increased gloss index values, more pronounced in the ventral region, which has better blood supply and higher temperature, compared to the dorsal coat, which is more exposed to adverse environmental conditions. However, it should be noted that the improved coat condition results from changes in the skin, hair follicles, and the structure of the coat itself. The improvement in coat quality may be associated with changes in nutritional status, gut microbiota activity, or immune-related mechanisms; however, markers of skin inflammation and hypersensitivity were not evaluated in the present study.

## 5. Conclusions

Supplementation of healthy senior dogs with a *Bacillus subtilis*-derived postbiotic improved apparent nutrient digestibility, promoted favorable fecal fermentation by increasing acetate, propionate, and total short-chain fatty acid concentrations while reducing fecal ammonia, and selectively remodeled the fecal microbiota through enrichment of bacterial genera associated with intestinal homeostasis and carbohydrate fermentation. These microbial changes were accompanied by enhanced humoral immunity, as evidenced by increased serum IgG concentrations, reduced circulating cholesterol and triglyceride levels, and improved coat characteristics, without adversely affecting hematological, biochemical, or trace mineral homeostasis. Collectively, these findings indicate that *Bacillus subtilis*-derived postbiotics may represent a promising functional dietary strategy for supporting gastrointestinal function, metabolic health, and immune competence in healthy senior dogs. Nevertheless, the relatively small sample size, short supplementation period, and evaluation of a single breed limit the generalizability of the present findings. Further long-term studies involving larger and more diverse canine populations, together with metabolomic and functional microbiome analyses, are warranted to clarify the underlying mechanisms and confirm the clinical relevance of these beneficial effects.

## Figures and Tables

**Table 1:** Ingredient composition of dry extruded food, %.

Poultry meal	22
Barley	28
Maize	9
Maize gluten meal	9
Rice	25.6
Potassium chloride	0.4
Salt	0.12
Vitamin/mineral pre-mixture *	0.305
Sunflower oil	5.5
Beef tallow	4.9
Determined Chemical Composition	
Dry matter, %	91.5
Ash, %	5.18
Acid hydrolyzed ether extract, %	11.20
Crude fiber, %	5.45
Crude protein, %	24.45
ME, kcal/kg **	3519
Total dietary fiber, %	15.44
Insoluble dietary fiber, %	10.28
Soluble dietary fiber, %	2.16
Calcium, %	1.4
Phosphorus, %	0.82

* Vitamin–mineral premixture (mg/kg): biotin 250 mg, folate 2 mg, pantothenic acid 20 mg, niacin 50 mg, riboflavin 8 mg, thiamine 4 mg, Vit. A 3.6 mg, Vit. B6 5 mg, Vit. B12 25 mg, cholecalciferol 125 mg, Vit. E 50 mg, Mn proteinate 70 mg, Zn proteinate 100 mg, Cu proteinate 14 mg, Mo proteinate 700 mg, Fe proteinate 70 mg, Iodine 1 mg, Co proteinate 350 mg, Se proteinate 140 mg, Mg oxide 35.000 mg. ** Calculated using the NRC [19] equations.

**Table 2:** MIC assay results.

Microorganism	24 °C	60 °C	75 °C
*Staphylococcus aureus* ATCC25923	0.004 mg/mL	0.004 mg/mL	0.004 mg/mL
*Clostridium perfringens* NCTC3180	0.002 mg/mL	0.002 mg/mL	0.002 mg/mL
*Escherichia coli* ATCC25922	>2 mg/mL	>2 mg/mL	>2 mg/mL
*Salmonella* Typhimurium ATCC14028	>2 mg/mL	>2 mg/mL	>2 mg/mL

**Table 3:** MBC assay results.

Microorganism	24 °C	60 °C	75 °C
*Staphylococcus aureus* ATCC25923	0.008 mg/mL	0.008 mg/mL	0.008 mg/mL
*Clostridium perfringens* NCTC3180	0.004 mg/mL	0.004 mg/mL	0.004 mg/mL

**Table 4:** Nutrient digestibility coefficients of the groups, % (Mean ± SEM).

	CON	POS1	POS2	Linear	Quadratic	Overall *p*-Value
DMD	83.9 ± 0.75 ^b^	86.3 ± 0.25 ^ab^	86.9 ± 0.42 ^a^	0.2	0.607	0.044
OMD	85.6 ± 0.65 ^b^	88.8 ± 0.26 ^ab^	89.4 ± 0.22 ^a^	0.193	0.629	0.037
CPD	76.9 ± 0.89 ^b^	80.5 ± 0.37 ^a^	81.1 ± 0.68 ^a^	0.028	0.184	0.008
EED	97.2 ± 0.09	96.4 ± 0.02	97.0 ± 0.09	0.51	0.56	0.095
CFD	51.4 ± 0.85 ^b^	59.5 ± 0.74 ^a^	63.1 ± 0.88 ^a^	0.01	0.300	0.001

DMD, dry matter digestibility; OMD, organic matter digestibility; CPD, crude protein digestibility; EED, ether extract digestibility; CFD, Crude fiber digestibility; CON, control group receiving no postbiotic supplementation; POS1, dogs receiving 0.3 mL/day of postbiotic derived from *Bacillus subtilis*; POS2, dogs receiving 0.6 mL/day of postbiotic derived from *Bacillus subtilis*; SEM, standard error of the mean; ^a,b^; Values in the same row that do not share the same superscript letter differ significantly (*p* < 0.05).

**Table 5:** Faecal quality and faecal fermentation metabolites of dogs in control and postbiotic groups (Mean ± SEM).

Parameters	CON	POS1	POS2	Linear	Quadratic	Overall *p*-Value
Faecal consistency score	2.75 ± 0.31	2.25 ± 0.23	2.23 ± 0.47	0.096	0.303	0.505
Faecal output (g/day)	204.02 ± 12	186 ± 18	195 ± 25	0.085	0.525	0.102
Faecal pH	7.15 ± 0.14 ^a^	6.81 ± 0.05 ^ab^	6.75 ± 0.04 ^b^	0.001	0.212	0.007
Faecal DM	35.90 ± 0.8	34.31 ± 1.8	35.49 ± 0.4	0.846	0.645	0.909
Faecal ammonia (µmol/g DM)	1.85 ± 0.6 ^a^	0.81 ± 0.8 ^b^	0.74 ± 0.3 ^b^	0.001	0.201	0.001
Acetate (µmol/g DM)	170.10 ± 38 ^b^	195.22 ± 25 ^a^	211.32 ± 45 ^a^	0.001	0.312	0.04
Propionate (µmol/g DM)	105.32 ± 18 ^b^	111.34 ± 11 ^b^	129.21 ± 12 ^a^	0.02	0.08	0.005
Butyrate (µmol/g DM)	20.22 ± 3	21.09 ± 4	22.56 ± 3	0.089	0.095	0.890
Isobutyrate (µmol/g DM)	3.31 ± 0.8	3.25 ± 0.8	3.37 ± 0.7	0.195	0.302	0.655
Isovalerate (µmol/g DM)	2.34 ± 0.1	2.24 ± 0.4	2.29 ± 0.3	0.175	0.165	0.452
Valerate (µmol/g DM)	0.65 ± 0.6	0.68 ± 0.8	0.64 ± 0.5	0.085	0.845	0.955
Total SCFAs (µmol/g DM)	273.52 ± 17 ^b^	318.23 ± 21 ^b^	331.67 ± 29 ^a^	0.001	0.325	0.035

CON, control group receiving no postbiotic supplementation; POS1, dogs receiving 0.3 mL/day of postbiotic derived from *Bacillus subtilis*; POS2, dogs receiving 0.6 mL/day of postbiotic derived from *Bacillus subtilis*; ^a,b^; Values in the same row that do not share the same superscript letter differ significantly (*p* < 0.05).

**Table 6:** Presence/absence-based alpha diversity indices of the fecal microbiota in dogs receiving the experimental diets (*n* = 7 per group).

Index	CON	POS1	POS2	Pooled SEM	ANOVA *p*-Value	Kruskal–Wallis *p*-Value	FDR-Adjusted *p*-Value	η^2^
Observed ASVs (*n*)	54.9 ᵃ	64.1 ᵃ	38.7 ᵇ	4.854	0.006	0.017	0.044	0.438
Observed genera (*n*)	7.3	9.3	6.9	0.805	0.103	0.102	0.205	0.224
Observed families (*n*)	7.7 ᵃ	9.6 ᵃ	6.7 ᵇ	0.621	0.014	0.022	0.056	0.377
Observed phyla (*n*)	4.3	4.3	4.0	0.294	0.733	0.711	0.791	0.034
Shannon index (genus level)	1.026	1.146	1.065	0.101	0.698	0.462	0.728	0.039
Simpson index (1 − D, genus level)	0.434	0.473	0.452	0.044	0.828	0.546	0.728	0.021
Pielou’s evenness (genus level)	0.529	0.513	0.566	0.034	0.543	0.791	0.828	0.066
Shannon index (phylum level)	1.161	1.017	1.124	0.041	0.061	0.036	0.162	0.267

^a,b^ Means within the same row with different superscript letters differ significantly (*p* < 0.05). Post hoc comparisons were performed using Tukey’s honestly significant difference (HSD) test when assumptions of normality and homogeneity of variance were met, and Dunn’s test with Benjamini–Hochberg adjustment when these assumptions were violated. SEM, pooled standard error of the mean; η^2^, effect size (eta squared); FDR, Benjamini–Hochberg false discovery rate-adjusted P-value. All diversity indices were calculated from presence/absence data.

**Table 7:** Beta diversity analysis of the fecal microbiota based on presence/absence data using PERMANOVA, ANOSIM, and PERMDISP (9999 permutations).

Distance Metric (Taxonomic Level)	Pseudo-F	R^2^	PERMANOVA *p*-Value	ANOSIM R	ANOSIM *p*-Value	PERMDISP F	PERMDISP *p*-Value
Jaccard (ASV)	1.343	0.130	<0.001	0.497	<0.001	5.799	0.007
Sørensen–Dice (ASV)	1.563	0.148	<0.001	0.497	<0.001	5.607	0.006
Jaccard (Genus)	2.905	0.244	<0.001	0.393	<0.001	0.817	0.354
Sørensen–Dice (Genus)	3.826	0.298	<0.001	0.393	<0.001	1.052	0.285
Jaccard (Family)	2.686	0.230	<0.001	0.292	<0.001	2.295	0.069

R^2^, proportion of the total variation in microbial community composition explained by dietary treatment; PERMANOVA, permutational multivariate analysis of variance; ANOSIM, analysis of similarities; PERMDISP, permutational analysis of multivariate dispersions. PERMANOVA and PERMDISP were performed using 9999 permutations.

**Table 8:** Pairwise PERMANOVA comparisons of fecal microbial community composition among treatment groups based on presence/absence data (9999 permutations). *p*-values were adjusted using the Benjamini–Hochberg false discovery rate (FDR) procedure within each distance metric.

Distance Metric (Taxonomic Level)	Comparison	Pseudo-F	R^2^	*p*-Value	FDR-Adjusted *p*-Value
Jaccard (ASV)	CON vs. POS1	1.416	0.106	<0.001	0.001
Jaccard (ASV)	CON vs. POS2	1.297	0.098	<0.001	<0.001
Jaccard (ASV)	POS1 vs. POS2	1.317	0.099	0.003	0.003
Sørensen–Dice (ASV)	CON vs. POS1	1.659	0.121	<0.001	<0.001
Sørensen–Dice (ASV)	CON vs. POS2	1.583	0.117	<0.001	<0.001
Sørensen–Dice (ASV)	POS1 vs. POS2	1.454	0.108	0.002	0.002
Jaccard (Genus)	CON vs. POS1	3.004	0.200	0.004	0.004
Jaccard (Genus)	CON vs. POS2	2.809	0.190	0.001	0.002
Jaccard (Genus)	POS1 vs. POS2	2.918	0.196	0.001	0.002
Sørensen–Dice (Genus)	CON vs. POS1	4.078	0.254	0.003	0.003
Sørensen–Dice (Genus)	CON vs. POS2	3.848	0.243	0.002	0.003
Sørensen–Dice (Genus)	POS1 vs. POS2	3.615	0.232	0.002	0.003
Jaccard (Family)	CON vs. POS1	3.875	0.244	<0.001	0.002
Jaccard (Family)	CON vs. POS2	2.079	0.148	0.035	0.035
Jaccard (Family)	POS1 vs. POS2	2.351	0.164	0.013	0.020

PERMANOVA, permutational multivariate analysis of variance; Pseudo-F, pseudo-F statistic obtained from PERMANOVA; R^2^, proportion of variation in microbial community composition explained by treatment; FDR, Benjamini–Hochberg false discovery rate-adjusted *p*-value. Pairwise PERMANOVA comparisons were performed using 9999 permutations. *p*-values were adjusted for multiple comparisons using the Benjamini–Hochberg false discovery rate (FDR) procedure separately within each distance metric. Statistical significance was declared at *p* < 0.05. Analyses were performed using presence/absence-based community data.

**Table 9:** Relative abundance (%) of the dominant bacterial phyla in the fecal microbiota of dogs receiving the experimental diets (*n* = 7 per group).

Phylum	CON (%)	POS1 (%)	POS2 (%)	ANOVA *p*-Value	Kruskal–Wallis *p*-Value	FDR-Adjusted *p*-Value
Firmicutes	23.51 ± 2.05	44.14 ± 5.04	32.74 ± 3.34	0.004	0.005	0.037
Bacteroidota	13.86 ± 2.04	3.24 ± 1.12	9.88 ± 3.96	0.034	0.013	0.045
Fusobacteriota	2.75 ± 0.71	2.83 ± 0.28	3.01 ± 0.28	0.201	0.063	0.146
Proteobacteria	0.29 ± 0.29	2.53 ± 0.76	2.62 ± 1.10	0.028	0.084	0.146
Actinobacteriota	3.93 ± 0.75	2.45 ± 1.06	2.02 ± 0.73	0.283	0.206	0.241
Campylobacterota	0.18 ± 0.18	0.00 ± 0.00	0.00 ± 0.00	0.387	0.368	0.368

Values are presented as mean ± SEM. FDR, Benjamini–Hochberg false discovery rate-adjusted *p*-value. Statistical significance was declared at *p* < 0.05.

**Table 10:** Relative abundance (%) of the major bacterial genera identified in the fecal microbiota of dogs receiving the experimental diets.

	Control	POS1	POS2	SD	SEM	Linear	Quadratic	Overall *p* Value
*Turicibacter*	3.92 ^b^	11.73 ^a^	11.44 ^a^	6.55	1.35	<0.001	0.85	0.001
*Fusobacterium*	5.65 ^b^	4.95 ^b^	9.49 ^a^	3.52	0.95	0.045	0.742	0.605
*Clostridium*	7.18	6.42	4.26	2.88	2.12	0.201	0.212	0.144
*Streptomyces*	0.02 ^c^	0.21 ^b^	6.58 ^a^	1.45	0.65	<0.001	0.338	0.669
*Segatella*	0.64 ^c^	4.82 ^b^	6.37 ^a^	2.45	1.82	<0.001	0.515	0.802
*Sarcina*	4.36 ^a^	0.11 ^c^	3.15 ^b^	2.66	2.02	0.009	0.053	0.155
*Bacteroides*	14.86 ^a^	2.05 ^b^	2.83 ^b^	4.26	2.15	0.011	0.682	0.385
*Ligilactobacillus*	0.04 ^b^	2.03 ^a^	2.69 ^a^	0.95	0.88	0.014	0.625	0.255
*Streptococcus*	1.32 ^b^	5.92 ^a^	2.47 ^b^	2.99	1.85	0.04	0.054	0.201
*Neisseria*	2.97	2.18	1.92	0.58	0.45	0.137	0.415	0.209
*Achromobacter*	8.51 ^a^	8.26 ^a^	1.63 ^b^	4.15	2.12	0.003	0.385	0.706
*Bartonella*	1.01	0.91	1.55	0.12	0.25	0.001	0.578	<0.001
*Terrisporobacter*	1.02	0.67	1.39	0.11	0.85	0.095	0.065	0.208
*Faecalibacterium*	0.47 ^c^	3.02 ^a^	1.08 ^b^	0.65	0.25	0.028	0.271	0.008
*Prevotella*	3.17 ^ab^	2.39 ^b^	3.99 ^a^	1.46	0.58	0.009	0.425	0.025
*Bifidobacterium*	0.005 ^c^	0.02 ^b^	0.74 ^a^	0.29	0.09	0.006	0.725	0.152
*Lactobacillus*	0.03	0.08	0.08	0.66	0.44	0.545	0.712	0.605
*Enterococcus*	0.02 ^c^	0.91 ^a^	0.2 ^b^	0.74	0.14	0.009	0.486	0.018

POS1, dogs receiving 0.3 mL/day of postbiotic derived from *Bacillus subtilis*; POS2, dogs receiving 0.6 mL/day of postbiotic derived from *Bacillus subtilis*; SD, standard deviation; SEM, standard error of the mean. Linear and Quadratic represent orthogonal polynomial contrast analyses evaluating dose-dependent responses to postbiotic supplementation. Different superscript letters (a–c) within the same row indicate significant differences among groups (*p* < 0.05). Only genera with a mean relative abundance ≥ 0.5% in at least one treatment group and/or a significant treatment effect (*p* < 0.05) are presented.

**Table 11:** Effects of postbiotic addition on hematological and biochemical parameters of dogs (mean ± SD).

	**Time (Day)**	***n***	**WBC,** **10^9^/L**	**NEU,** **10^9^/L**	**LYM,** **10^9^/L**		**MON,** **10^9^/L**	**EOS,** **10^9^/L**		**RBC, 10^12^/L**	**HGB,** **g/dL**	**HCT,** **%**
CON	0	7	8.9 ± 2.05	5.4 ± 1.2	2.9 ± 0.8		0.26 ± 0.08	0.2 ± 0.07		6.5 ± 0.6	16.6 ± 1.8	45.3 ± 4.6
	28	7	9.8 ± 2.08	5.7 ± 3.3	2.8 ± 0.9		0.29 ± 0.1	0.3 ± 0.1		6.6 ± 0.7	16.1 ± 1.9	44.9 ± 4.5
Wilcoxon		*p*	0.238	0.612	0.098		0.068	0.078		0.799	0.236	0.058
POS1	0	7	8.9 ± 1.5	5.8 ± 1.4	2.5 ± 1.1		0.35 ± 0.2	0.2 ± 0.8		6.4 ± 0.9	15.6 ± 2.3	42.9 ± 5.8
	28	7	10.7 ± 4.2	6.8 ± 2.7	3.1 ± 1.6		0.43 ± 0.2	0.3 ± 1.0		6.8 ± 0.4	16.7 ± 0.9	43.9 ± 2.6
Wilcoxon		*p*	0.237	0.499	0.176		0.398	0.115		0.237	0.398	0.499
POS2	0	7	8.6 ± 1.6	5.5 ± 1.7	2.5 ± 0.3		0.2 ± 0.1	0.3 ± 0.1		6.5 ± 0.4	15.9 ± 1.5	44.3 ± 5.1
	28	7	8.3 ± 1.3	4.7 ± 0.9	2.8 ± 0.6		0.4 ± 0.1	0.2 ± 0.1		6.7 ± 0.5	15.9 ± 1.6	40.6 ± 3.8
Wilcoxon		*p*	0.866	0.499	0.091		0.018	0.075		0.237	0.799	0.063
CON	28	7	9.8 ± 2.08	5.7 ± 3.3	2.8 ± 0.9		0.29 ± 0.1	0.3 ± 0.1		6.6 ± 0.7	16.1 ± 1.9	44.9 ± 4.5
POS1	28	7	10.7 ± 4.2	6.8 ± 2.7	3.1 ± 1.6		0.43 ± 0.2	0.3 ± 1.0		6.8 ± 0.4	16.7 ± 0.9	43.9 ± 2.6
POS2	28	7	8.3 ± 1.3	4.7 ± 0.9	2.8 ± 0.6		0.4 ± 0.1	0.2 ± 0.1		6.7 ± 0.5	15.9 ± 1.6	40.6 ± 3.8
		*p*	0.231	0.116	0.615		0.554	0.252		0.350	0.456	0.350
Reference ranges			5.9–16.6	3.6–12.3	0.8–12.3		0.2–0.8	0.14–1.97		5.1–8.5	11–19	33–56
	**Time (Day)**	***n***	**MCV,** **fL**	**MCH,** **pg**	**MCHC,** **g/dL**	**IgE,** **ng/mL**	**IgG,** **mg/mL**	**BUN,** **mg/dL**	**CRE,** **mg/dL**	**ALB,** **g/dL**	**CHOL,** **mg/dL**	**TRIG,** **mg/dL**
CON	0	7	68.6 ± 3.1	25.1 ± 1.2	36.6 ± 0.32	1469.9 ± 44	2.2 ± 0.6	14.7 ± 3.7	1.2 ± 0.3	2.9 ± 0.3	268 ± 34	49 ± 11
	28	7	63.5 ± 2.7	24.7 ± 0.8	38.2 ± 1.5	1441.9 ± 49	2.3 ± 0.4	15.3 ± 2.6	0.9 ± 0.1	3 ± 0.4	259 ± 61	53 ±14
Wilcoxon		*p*	0.058	0.178	0.090	0.866	0.069	0.866	0.101	0.340	0.398	0.553
POS1	0	7	67.5 ± 2.2	24.5 ± 0.8	36.3 ± 0.5	603.6 ± 18	1.9 ± 0.3	11.1 ± 5.4	1.2 ± 0.2	2.8 ± 0.4	254 ± 38	65 ± 22
	28	7	64.1 ± 2.5	24.4 ± 0.9	38.1 ± 0.9	818.3 ± 33	3.3 ± 0.7	12.1 ± 5.8	0.9 ± 0.3	3.1 ± 0.4	234 ± 14	42 ± 11
Wilcoxon		*p*	0.064	0.233	0.018	0.063	0.023	0.075	0.026	0.279	0.035	0.028
POS2	0	7	67.7 ± 3.6	24.3 ± 1.1	35.9 ± 0.7	1051.8 ± 27	2.1 ± 0.5	13.2 ± 1.5	1.4 ± 0.2	2.8 ± 0.3	238 ± 36	70 ± 9.4
	28	7	60.3 ± 3.5	23.7 ± 0.9	39.4 ± 1.9	1322.7 ± 30	2.9 ± 0.9	12.9 ± 1.1	1.1 ± 0.1	2.6 ± 0.8	207 ± 17	44 ± 7.8
Wilcoxon		*p*	0.018	0.134	0.028	0.091	0.018	0.063	0.034	0.397	0.045	0.022
CON	28	7	63.5 ± 2.7 ^a^	24.7 ± 0.8	38.2 ± 1.5			15.3 ± 2.6	0.9 ± 0.1	3 ± 0.4	259 ± 61 ^a^	53 ± 14 ^a^
POS1	28	7	64.1 ± 2.5 ^a^	24.4 ± 0.9	38.1 ± 0.9			12.1 ± 5.8	0.9 ± 0.3	3.1 ± 0.4	234 ± 14 ^b^	42 ± 11 ^b^
POS2	28	7	60.3 ± 3.5 ^b^	23.7 ± 0.9	39.4 ± 1.9			12.9 ± 1.1	1.1 ± 0.1	2.6 ± 0.8	207 ± 17 ^c^	44 ± 7 ^b^
Bonferroni		*p*	<0.001	0.239	0.210			0.065	0.083	0.643	0.003	0.001
Reference ranges			60–76	20–27	30–38			5.6–28	5.5–8.5	60–75	125–270	20–112

CON, control group receiving the basal diet without postbiotic supplementation; POS1, dogs receiving 0.3 mL/day of a postbiotic derived from *Bacillus subtilis*; POS2, dogs receiving 0.6 mL/day of the same postbiotic. *n*, number of animals per group. WBC, white blood cell count; NEU, neutrophils; LYM, lymphocytes; MON, monocytes; EOS, eosinophils; RBC, red blood cell count; HGB, hemoglobin; HCT, hematocrit; MCV, mean corpuscular volume; MCH, mean corpuscular hemoglobin; MCHC, mean corpuscular hemoglobin concentration; IgE, immunoglobulin E; IgG, immunoglobulin G; BUN, blood urea nitrogen; CRE, creatinine; ALB, albumin; CHOL, total cholesterol; TRIG, triglycerides. Different superscript letters (a–c) within the same row indicate significant differences among groups (*p* < 0.05).

**Table 12:** Changes in serum trace mineral concentrations of dogs in control and postbiotic-supplemented groups (Mean ± SEM).

	Time (Day)	*n*	Co, µg	Cu, µg	Cr, ppm	Fe, µg	Zn, µg	Mn, mg	Se, µmol/L
CON	0	7	0.53 ± 0.07	120.6 ± 2.25	2771.4 ± 82.9	168.8 ± 6.2	260.8 ± 4.4	0.77 ± 0.16	2.78 ± 0.05
	28	7	0.51 ± 0.01	118.3 ± 1.32	2778.6 ± 70.1	166.1 ± 5.2	260.7 ± 5.2	0.75 ± 0.15	2.84 ± 0.04
*T*-test		*p*-value	0.101	0.118	0.915	0.420	0.959	0.075	0.175
POS1	0	7	0.55 ± 0.05	119.3 ± 3.1	2601.1 ± 45.2	141.7 ± 7.6	251.6 ± 3.4	0.8± 0.08	2.42 ± 0.07
	28	7	0.52 ± 0.01	118.5 ± 0.9	2801.4 ± 71.1	161.6 ± 5.3	257.3 ± 3.6	1.2 ± 0.03	2.92 ± 0.05
*T*-test		*p*-value	0.380	0.805	0.064	0.012	0.272	0.025	0.002
POS2	0	7	0.50 ± 0.01	113.1 ± 2.2	2646.8 ± 39.3	157.1± 3.9	250 ± 3.6	0.94 ± 0.03	2.66 ± 0.06
	28	7	0.53 ± 0.01	118.3 ± 1.5	2822.9 ± 52.6	173.2 ± 3.5	254.7 ± 3.1	1.09 ± 0.02	2.77 ± 0.03
*T*-test		*p*-value	0.126	0.021	0.010	0.043	0.143	0.008	0.098

CON: control group receiving the basal diet without postbiotic supplementation; POS1: dogs receiving 0.3 mL/dog/day of a *Bacillus subtilis*-derived postbiotic; POS2: dogs receiving 0.6 mL/dog/day of a *Bacillus subtilis*-derived postbiotic. Co: cobalt; Cu: copper; Cr: chromium; Fe: iron; Zn: zinc; Mn: manganese; Se: selenium. Data are presented as mean ± standard error of the mean (SEM) (*n* = 7 per group).

## Data Availability

The raw data supporting the conclusions of this article will be made available by the authors on request.

## Abbreviations

The following abbreviations are used in this manuscript:

ATTD	Apparent Total Tract Digestibility
BUN	Blood Urea Nitrogen
CA	Crude Ash
CF	Crude Fiber
CFD	Crude Fiber Digestibility
CON	Control Group
CP	Crude Protein
CPD	Crude Protein Digestibility
DM	Dry Matter
DMD	Dry Matter Digestibility
EE	Ether Extract
ELISA	Enzyme-Linked Immunosorbent Assay
FOS	Fructooligosaccharides
GU	Gloss Units
ICP-MS	Inductively Coupled Plasma Mass Spectrometry
IDF	Insoluble Dietary Fiber
IgE	Immunoglobulin E
IgG	Immunoglobulin G
ISAPP	International Scientific Association of Probiotics and Prebiotics
MAMPs	Microbe-Associated Molecular Patterns
MBC	Minimum Bactericidal Concentration
MCV	Mean Corpuscular Volume
MCHC	Mean Corpuscular Hemoglobin Concentration
ME	Metabolizable Energy
MIC	Minimum Inhibitory Concentration
MLN	Mesenteric Lymph Nodes
MOS	Mannan-Oligosaccharides
NRC	National Research Council
OMD	Organic Matter Digestibility
POS1	Postbiotic-Supplemented Group Receiving 0.3 mL/day
POS2	Postbiotic-Supplemented Group Receiving 0.6 mL/day
SCFA	Short-Chain Fatty Acid
SDF	Soluble Dietary Fiber
SEM	Scanning Electron Microscopy
TDF	Total Dietary Fiber
